# Evidence of infectious disease, trauma, disability and deficiency in skeletons from the 19th/20th century correctional facility and asylum «Realta» in Cazis, Switzerland

**DOI:** 10.1371/journal.pone.0216483

**Published:** 2019-05-08

**Authors:** Christine Cooper, Bernd Heinzle, Thomas Reitmaier

**Affiliations:** 1 Archaeological Service of the Canton of Grisons, Chur, Switzerland; 2 Department of Archaeology, Office of Culture, Triesen, Principality of Liechtenstein; University of Delaware, UNITED STATES

## Abstract

As a reaction to widespread poverty, a system of coercive welfare developed in Switzerland during the 19^th^ century. Poverty was often thought to result from an individual’s misconduct rather than from structural, economic or political circumstances. People whose lifestyle deviated from the desired norm or who were unable to make a living for themselves were subjected to so-called administrative detention at institutions such as workhouses and poorhouses. The excavation of the cemetery of the correctional facility/workhouse and asylum «Realta» in Cazis offered the opportunity to gain insight into the living conditions of a marginalized group of people and to shed light on aspects of coercive welfare that have hardly been addressed in historical studies. A comprehensive study of pathological alterations was used to assess possible physical causes and effects of administrative detention. Skeletal samples from regular contemporaneous cemeteries provided data for the general population and thus allowed us to detect peculiarities in the «Realta» assemblage. Possible cases of Stickler Syndrome, microcephaly, congenital syphilis, endemic hypothyroidism and disabilities secondary to trauma may have been the reason for the affected individuals’ institutionalisation. The high prevalence of tuberculosis was linked to the socioeconomic status and the living conditions at the facility. Several cases of scurvy and osteomalacia may have resulted from various risk factors such as poverty, alcoholism, mental illness or institutionalisation. The fracture rates, especially of ribs, were extremely high. A large proportion of the fractures were incompletely healed and most likely occurred during detention due to interpersonal violence. Underlying diseases further contributed to the high fracture rates. This first study on skeletons from an institution of administrative detention in Switzerland demonstrated how pre-existing health conditions and the socioeconomic background contributed to the chance of being detained, and how detention led to further deterioration of health.

## Introduction

### A system of coercive welfare in Switzerland

Within the modern Swiss federal state, the treatment of socio-political issues was the responsibility of individual cantons and municipalities from 1848 onwards. This included the handling of poverty, which was considered a problem for the entire society in the 19^th^ century. The cantonal «Armenordnungen» («Ordinances for the Poor») from 1839, 1840 and 1857 not only regulated the material support of the needy, but also contained educative and repressive/punitive measures [1,2,3,4]. The communities were put in charge of caring for citizens who were impoverished for reasons outside of their own control. This could be a significant burden on communities in structurally weak areas, because in some cases up to a quarter of their citizens were in need of assistance. One way to limit the cost was to provide one-time financial support for a person to emigrate overseas. Furthermore, persons could be admitted to institutions such as poorhouses, nurseries or workhouses. This so-called «Administrative Versorgung» (administrative detention) was not decided by the municipality itself but by the «Commission for the Poor», and from 1857 onward by the cantonal guardianship authority. This authority could decide a person’s admission to an institution on its own or when this measure was suggested by the municipality or the family. The decision also needed to be approved by the cantonal government. Since the canton was involved in this type of administrative care, the costs of accommodation were shared [4].

This interference with personal freedom was legitimated by the need to protect society from troublemakers and potential criminals in most cases, but sometimes also with the necessity to care for and protect those affected [5]. In most cases, poverty was thought to result from an individual’s misconduct rather than from structural, economic or political circumstances. These ideas lead to a social and legal devaluation of the poor, and consequently to the withdrawal of the right to vote, settle and marry, for example. Lifestyles that deviated from the desired norm and were therefore subject to corrective measures were primarily classified with two strongly sex-specific terms: «arbeitsscheu» (work-shy) and «liederlich» (dissolute). Men were often categorized as «work-shy» which referred to an unsatisfactory working morale and the lack of continuous gainful employment. Women accused of transgressions against sexual and moral norms, such as prostitution or extramarital sex and births, were branded as «dissolute» [1,2,3,4]. Those accused of being work-shy or dissolute were considered unworthy of support but were supposed to be educated to work or at least disciplined by the threat of institutionalisation. For this purpose, forced labour institutions were established in many cantons [5]. Detention at such an institution was legally based on two different approaches: The first was of repressive-punitive nature, while the second aimed at exerting an educative-disciplinary influence with the goal of correcting the person’s social behaviour. However, administrative detention was not necessarily the result of previous misconduct or impoverishment but could also be applied as a preventive measure when the social and moral conduct of an individual was feared to deteriorate.

From an early stage of the administrative detention system, so-called «lunatics» were also detained at institutions. Later, the additional category of «drunkards» stigmatised alcohol abuse as another form of social misconduct. The facilities of administrative detention were often melting pots for various groups of people, some of whom were too old and ill or physically and mentally unable to care for themselves and were not provided for by their families or communities.

Until the 1910s, those placed in administrative care could not appeal against the decision. Coercive welfare measures were applied in Switzerland until 1981 and have increasingly become the subject of socio-political debates and research in recent years [1,2,3,4,5,6,7,8,9].

### Administrative detention in the Canton of Grisons

**Fig 1:**
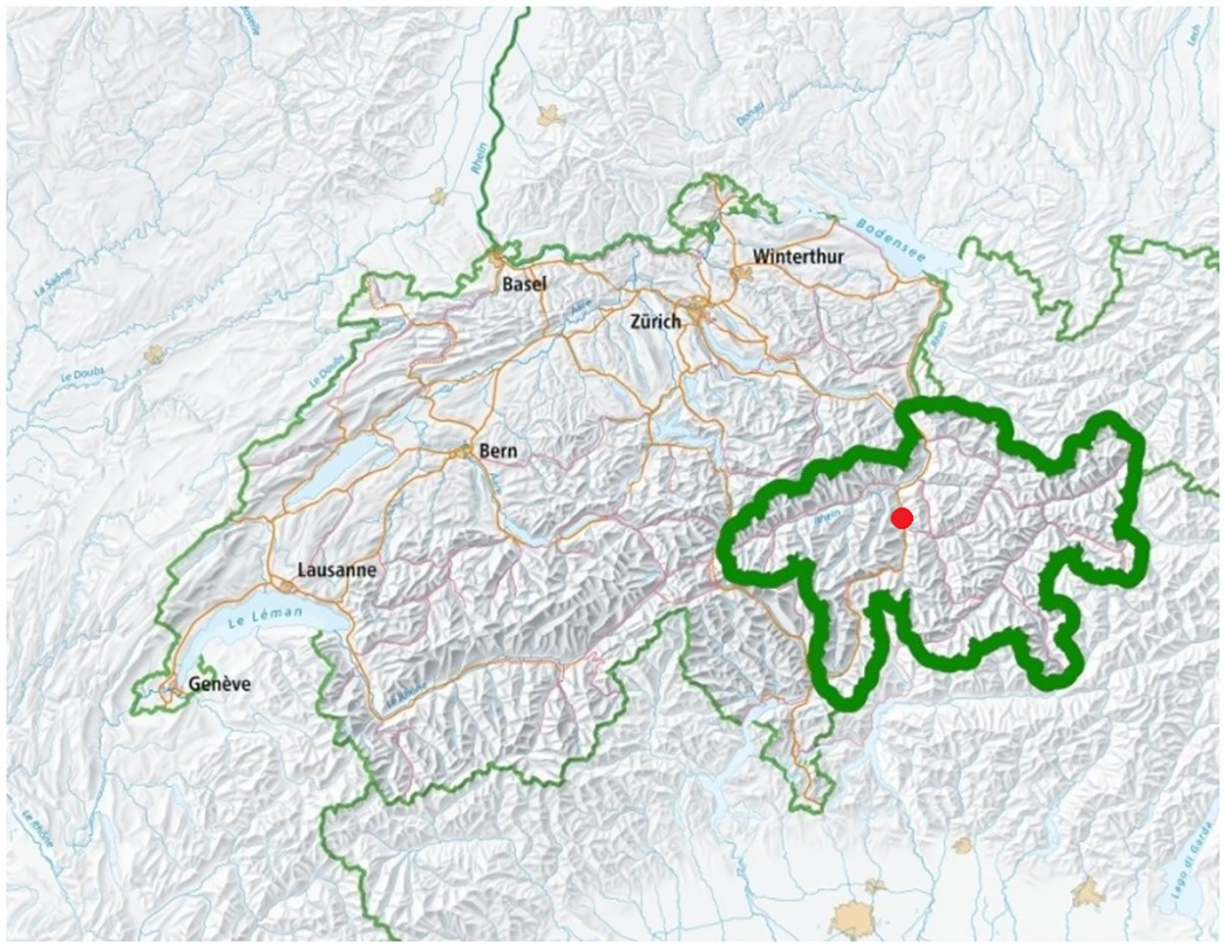
Map of Switzerland with the Canton of Grisons (thick green line) and the location of Cazis/Realta (red).

The «Forced Labour Institution Fürstenau», which opened in 1840, was the first institution of this type in the Canton of Grisons. It was replaced in 1855 by the newly erected «Correctional Institution Realta», also known as «Realta Correctional Centre and Asylum» or simply «Realta» near the village Cazis (Fig 1). The institution comprised two wards: one for those who were there for the purpose of correcting their behaviour (the workhouse), the other for the so-called lunatics. The «Realta», like its predecessor, was able to accommodate about 50 people, but offered many more occupational opportunities. The inmates worked at the institution-owned agricultural and craft businesses or were occasionally sent to work for private persons outside the institution (for example, in the hay harvest). Another major field of activity was the correction of the nearby Rhine River that was conducted by the canton. The inmates were paid by the canton at a fixed rate for their work. However, this amount was offset by the higher feeding allowance which the municipalities had to pay for the person’s accommodation at the institution [4].

Between 1855 and 1918, approximately 400–600 people were housed at the facility. So-called lunatics accounted for just over half of the detainees in the early decades [4]. Among them were seriously ill, bedridden persons with mental impairments. Even the institution’s director bemoaned the inhumane conditions and poor provision for these persons in his institution in the 1880s [10,11].

During this period, at least 110 inmates died during their stay, about 100 of them between 1855 and 1892. The causes of death were reported for 71 individuals in the institution’s records. Accidents led to death in about 2% of the cases and suicides in about 7%. For the remaining persons, there was often the mention of an indeterminate illness as the cause of death [12,13]. As prescribed by the institutional regulations from 1858, the deceased were normally buried in the institution’s own cemetery [14]. In only a few cases is there clear evidence that the bodies were transported back home. The last entry for a person who died and was probably buried at the institution dates from 1915. A new asylum was then built in the immediate vicinity of the «Realta». It had its own burial ground from 1919 onward [15,16] which most likely replaced the already overcrowded «Realta» cemetery. In addition, the new regulations of the institutions from 1917 explicitly allowed for the deceased to be brought home [17]. The «Correctional Institution Realta» was renamed to «Work Education Institution» in 1941 and converted to a custody institution in 1965. Since 1986 it has served as a prison.

### The «Realta» cemetery in Cazis

The construction of a new prison just north of the former correctional facility began in 2016. The former «Realta» cemetery was no longer visible at the surface but was located on the basis of historical maps and plans as well as oral tradition. Large parts of it would have been destroyed during the construction project, and for this reason it was excavated by the Archaeological Service of the Canton of Grisons [18]. Beforehand, information about the cemetery and those buried there was collected by means of archival research. This allowed us to identify 102 persons who were certainly or most likely buried in this cemetery, as well as three others who may have been interred there but could not be verified. Their average duration of detention before death was 48 months for males and 46 months for females. Four individuals spent less than a month at the institution before their death. At the other end of the spectrum were eleven persons who had been at the institution for more than ten years, with a maximum stay of around 30 years. Eighty individuals had been at the institution for more than six months before they died [19]. No plan of the cemetery’s structure or the location of identified individual graves in the cemetery exists.

**Fig 2:**
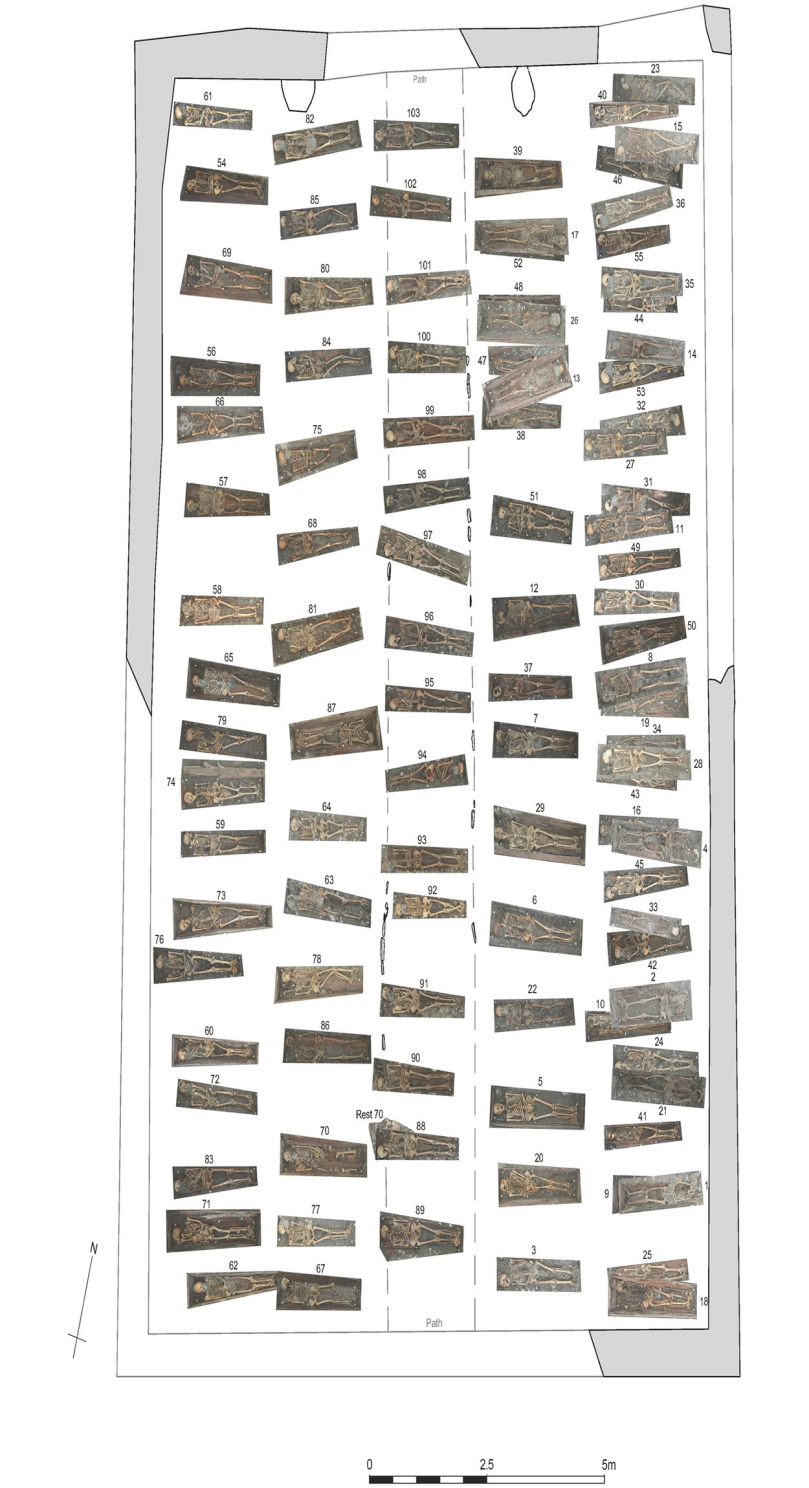
Location of the individual graves within the cemetery (rectified images). Only parts of the cemetery wall were found.

After geophysical investigations, the excavation took place from May to July 2016. In total, 103 individuals were excavated, documented and recovered. DNA samples as well as soil samples for palaeoparasitological studies were taken from all individuals and are available for future investigations. The cemetery itself was walled and originally had a central path and thus two separate burial areas. It covered about 13 m x 19 m and showed a slight slope to the north and east. The capacity of the cemetery was insufficient for the number of deceased, and thus only 67 of the 103 tombs were found in two rows each in the originally designated funerary areas. When these were full, the original path was used for 16 graves, and finally another 20 individuals were buried over the existing graves in the eastern burial area (Fig 2). The deeper graves were respected and spared as much as possible. No grave markings were found. Traces of wooden coffins were discovered in all graves, and in some cases, the caskets and lids were almost completely preserved. In addition to clothing and costume remains such as buttons, earrings and finger rings, there were personal items which had been left with the deceased person: a Jew’s harp, a religious book, a rosary or a glass vial. Five graves contained puparia which showed that fly larvae had been present on the body.

Most of the deceased were buried with their heads in the west, meaning that they were liturgically facing the east. As a tendency, the number of individuals with this orientation decreased over time. The historical sources as well as the original layout of the cemetery suggested separate funerary areas for Protestant and Catholic inmates. However, it was possible to approximately reconstruct the burial sequence of the last 35 individuals and to synchronise it with the historical and anthropological data available so far, but the findings from this latest burial phase spoke against a denominational separation. All in all, the archaeological findings showed a burial consistent with cultural traditions.

### State of research

The human remains from several cemeteries of 19^th^/20^th^ century institutions like poorhouses, workhouses and mental asylums in the United States and Europe have been excavated and studied anthropologically with different focuses [20,21,22,23,24,25,26,27,28,29,30]. An example from Central Europe is the cemetery of a psychiatric hospital in Hall (Austria) which was suspected to contain remains of possible victims of «wild euthanasia» (decentralized killing of people with disabilities) in the Nazi era [31]. In Switzerland, only one other cemetery of an administrative detention facility—the poorhouse in Riggisberg—has been excavated, but the examination of the skeletons is still ongoing [32,33]. The legal aspects, institutional developments and procedures of the Swiss system have been studied for several cantons, including Grisons [1,2,3,4,9]. While several case studies within this research paint a vivid picture of the fates of selected detainees, little is known about the physical causes and effects of life at such facilities.

### Aim of the study

The skeletal assemblage offered the opportunity to examine the anthropological and palaeopathological parameters that may complement the information from written records. One aim of this study was the identification of pathologies and the possible associated physical or mental impairments that may have led to the affected individuals’ institutionalisation, thus specifying vague contemporaneous terms like “insane”. Another focus was an assessment of the living conditions of this marginalized group of people. A comparative approach was chosen for this purpose. The findings were compared to data from contemporaneous skeletal samples from regular rural and urban cemeteries, and the observed similarities and differences were used to identify peculiarities in this assemblage that may shed light on the physical effects of administrative detention.

## Materials and methods

### Material

The material comprised all 103 skeletons that were recovered during the excavation at Cazis/Realta. Each of them was first examined on-site. In a next step, all bones were washed, followed by a comprehensive examination in the lab.

### Methods

All necessary permits were obtained for the study, which complied with all relevant regulations. This study deals with archaeological skeletal material. The Archaeological Service of the Canton of Grisons (under the directorship of Dr. T. Reitmaier) commissioned and licensed the archaeological fieldwork and the scientific analysis of the human skeletal remains based on the cantonal law for monuments and sites from 2010 ("Kantonales Natur- und Heimatschutzgesetz"). The remains date to ca. 1858–1917. A full identification of individual remains has not been possible and living family members are not known. An attempt to identify family members has not been made and is not planned, as has been agreed upon with the responsible political representative (M. Jäger, cantonal office of cultural affairs/EKUD). The remains are stored at the archive of the Archaeological Service of the Canton of Grisons in Chur, Switzerland (excavation number 52785, graves 1–103. The inventory list corresponds to the information given in the supporting information).

#### Bone count

All preserved bones were counted. The nasal bones, zygomatic and maxilla of each side were counted as one unit termed “facial bones”, as were the cervical, thoracic and lumbar vertebrae as well as the bones of the hand and foot. Each bone or unit was assigned a value of 0 (0–30% preserved), 0.5 (30–70% preserved) or 1 (70–100% preserved). Vertebrae and ribs were also counted individually. The number of ribs was determined by counting the vertebral ends, and a vertebral body was considered present when it was at least 50% preserved.

#### Determination of sex and age

Sex determination was based on sexually dimorphic traits of the pelvis and the skull, but general robustness of bones and joints was also considered [34,35]. The age of juveniles was estimated using the development and eruption of teeth [36,37] as well as the epi- and apophyseal union of postcranial bones [38]. The markers used for aging adults included the sternal clavicle [39], the pubic symphysis [34,40] and the auricular surface [41], the rib ends [42,43] and the closure of the cranial sutures [34]. Based on the arithmetic mean of the age range, every individual was assigned to one of the following age classes (years): 14–19.9, 20–29.9, 30–39.9, 40–49.9, 50–59.9, 60-x.

#### Pathological alterations and trauma

All bones were examined macroscopically for pathological alterations. Periosteal lesions were classified as active (woven or unremodeled) or healed (sclerotic or remodeled) [44,45].

Prevalence of new bone formation, lytic lesions and trauma was calculated per individual and per bone/unit. Individuals in which the respective skeletal element was not preserved or was not assessable were excluded from the calculation.

The standard criteria for timing traumatic lesions were applied to differentiate between antemortem, perimortem and postmortem trauma. Antemortem lesions are characterised by signs of healing. Perimortem lesions exhibit features indicative of the elasticity of living or freshly deceased bone, and their staining and decomposition do not differ from that of the surrounding bone. The edges of post-mortem breakage, in contrast, are usually lighter in colour than the surrounding bone, their decomposition is less pronounced, and the breakage does not resemble the pattern found in perimortem fractures [45,46,47,48,49,50,51]. To determine which traumata may have been sustained during detention, an attempt was made to narrow down the timing of antemortem injuries (Table 1). The duration of fracture healing depends on many factors such as age, affected bone, fracture type, diet and the general state of health [52,53,54]. The first macroscopically visible sign of healing is pitting of the bone around the fracture site, indicating osteoclastic activity, and blunting of the edges [51]. This is followed by slight formation of woven bone at the fracture site. The first signs cannot be seen before 1–2 weeks after the traumatic event [51,53,55]. Newly formed callus is irregular and raised above the bone surface [46]. By the sixth week, a calcified callus is present. The remodelling of the callus to lamellar bone can take up to six weeks in children and up to six months or longer in older adults [50,56,57].

**Table 1:** Criteria for the identification of different stages of fracture healing.

Stage	Macroscopic signs of healing	Approximate timing
1	None	<2 weeks before death
2	Pitting, blunting of edges, new bone formation	2–6 weeks before death
3	Calcified callus (woven bone)	6 weeks—ca. 6 months before death
4	Remodeled	>6 months before death

#### Statistics

Two-tailed Fisher exact tests were used to test the statistical significance of observations that produced categorical data. Results with P<0.05 were deemed significant.

#### Reference groups and archival data

Reference groups were chosen based on geographical proximity, similar dating and the availability of systematically recorded palaeopathological data. Two rural and two urban cemeteries from Switzerland and the Principality of Liechtenstein fulfilled the requirements (Table 2). To ensure consistency, the data of children from the reference groups were excluded when possible.

**Table 2:** The reference groups used in this study.

Site	Country	Total number of individuals	Dating	Reference
**Bern (Bundesgasse, Holzwerkhof)** Urban cemetery, higher social class	Switzerland	318	18^th^/19^th^ century	[58]
**Bern (Sidlerstrasse, Grosse Schanze)** Urban cemetery, lower social class	Switzerland	124	18^th^/19^th^ century	[58]
**Zweisimmen (Kirchgasse, Group B)** Rural cemetery	Switzerland	69	17^th^-19^th^ century	[59]
**Eschen (Friedhofserweiterung)** Rural cemetery	Liechtenstein	86	18^th^-20^th^ century	Cooper, unpublished

The available archival records included admission and egression registers, diaries, church registers and letters. From these, information about sex, age, duration of detention and place of burial was obtained [19]. These results were only disclosed after the completion of all anthropological examinations.

## Results

### Sex and age

A presumptive sex determination was possible for 99 individuals. The remaining four individuals exhibited ambiguous features, were juveniles or were very poorly preserved so that they were sexed as probable male or probable female. In the following considerations probable males/females were included in the male/female groups. The sample thus contained 68 (66%) male and 35 (34%) female individuals.

The determination of age at death revealed that all age classes from 14–19.9 to 60-x years were represented, with a predominance of individuals in the age class 40–59.9 years. The age distribution of males and females was very similar (Table 3).

**Table 3:** Age and sex distribution of the skeletal assemblage.

Age class (years)	Male		Female		Total	
	N	%	N	%	N	%
14–19.9	1	1.5	1	2.9	2	1.9
20–29.9	4	5.9	1	2.9	5	4.9
30–39.9	6	8.8	5	14.3	11	10.7
40–49.9	17	25.0	11	31.4	28	27.2
50–59.9	25	36.8	13	37.1	38	36.9
60-x	15	22.1	4	11.4	19	18.4
Total	68	100	35	100	103	100

**Fig 3:**
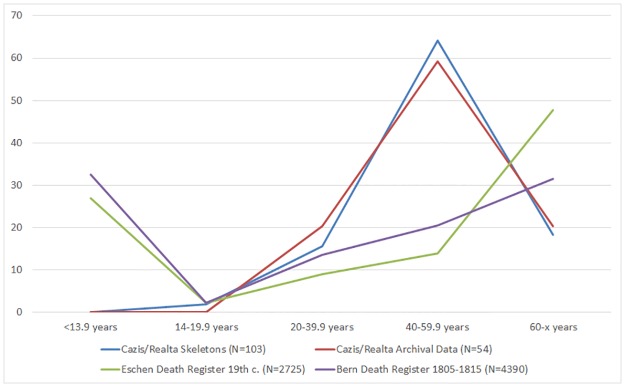
Age distributions (%) in the skeletal assemblage, archival records and two 19^th^ century death registers.

A comparison with the data of 54 persons, for whom the age at death was noted in the archival records, as well as with data from 19th century death registers from Bern [60] and Eschen [61], revealed that the age distributions obtained from the skeletons and from the institution’s archival records were almost identical. The death register data, which provides a realistic picture of the composition of the deceased in the general population during this period, was characterised by a relatively high proportion of children and the highest number of adult deaths above 60 years of age. In the skeletal assemblage from Cazis/Realta, however, children were not present, and most adult deaths were recorded between 40 and 60 years of age (Fig 3).

### Proliferative and lytic lesions

#### Cranial lesions

Ectocranial new bone formation was seen in ten individuals. The locations of the changes are summarised in Table 4.

**Table 4:** Distribution of ectocranial new bone formation.

Grave number	Sex	State of lesions	Cranial vault	Orbits	Zygomatic	Maxilla	Sphenoid	Mandible outside	Mandible inside (around Fossa submandibularis)	Long bones
47	Female	active				X	X			
49	Female	active						X		
59	Male	active							X	
61	Male	active		X	X	X	X	X	X	
67	Female	active							X	
69	Male	active							X	
75	Female	active	X							
76	Male (Fig 4)	partly healed	X		X	X	X	X		
90	Male	active		X						X
102	Female (Fig 5)	active		X				X		

**Fig 4:**
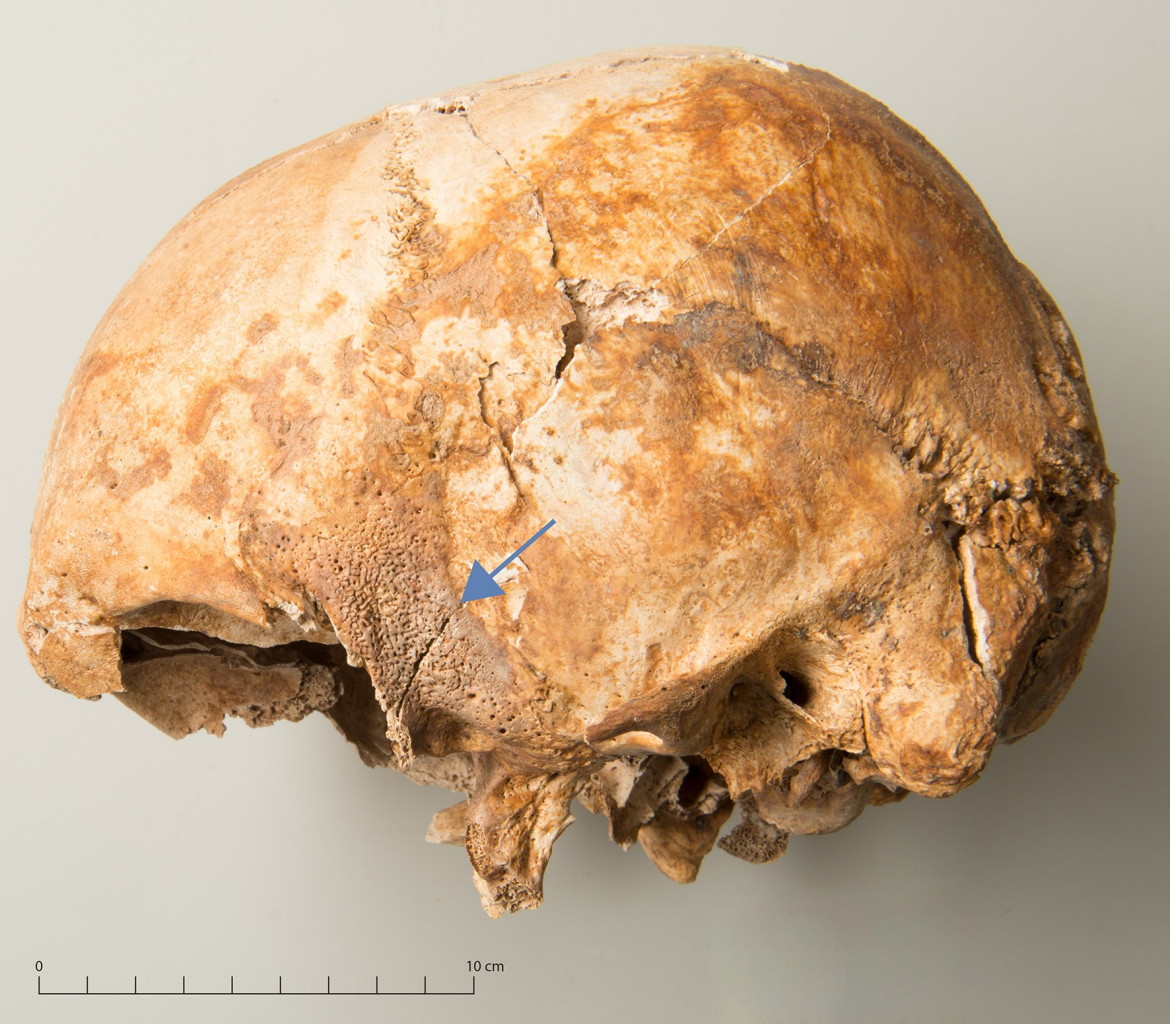
New bone formation in the greater wing of the sphenoid (g76).

**Fig 5:**
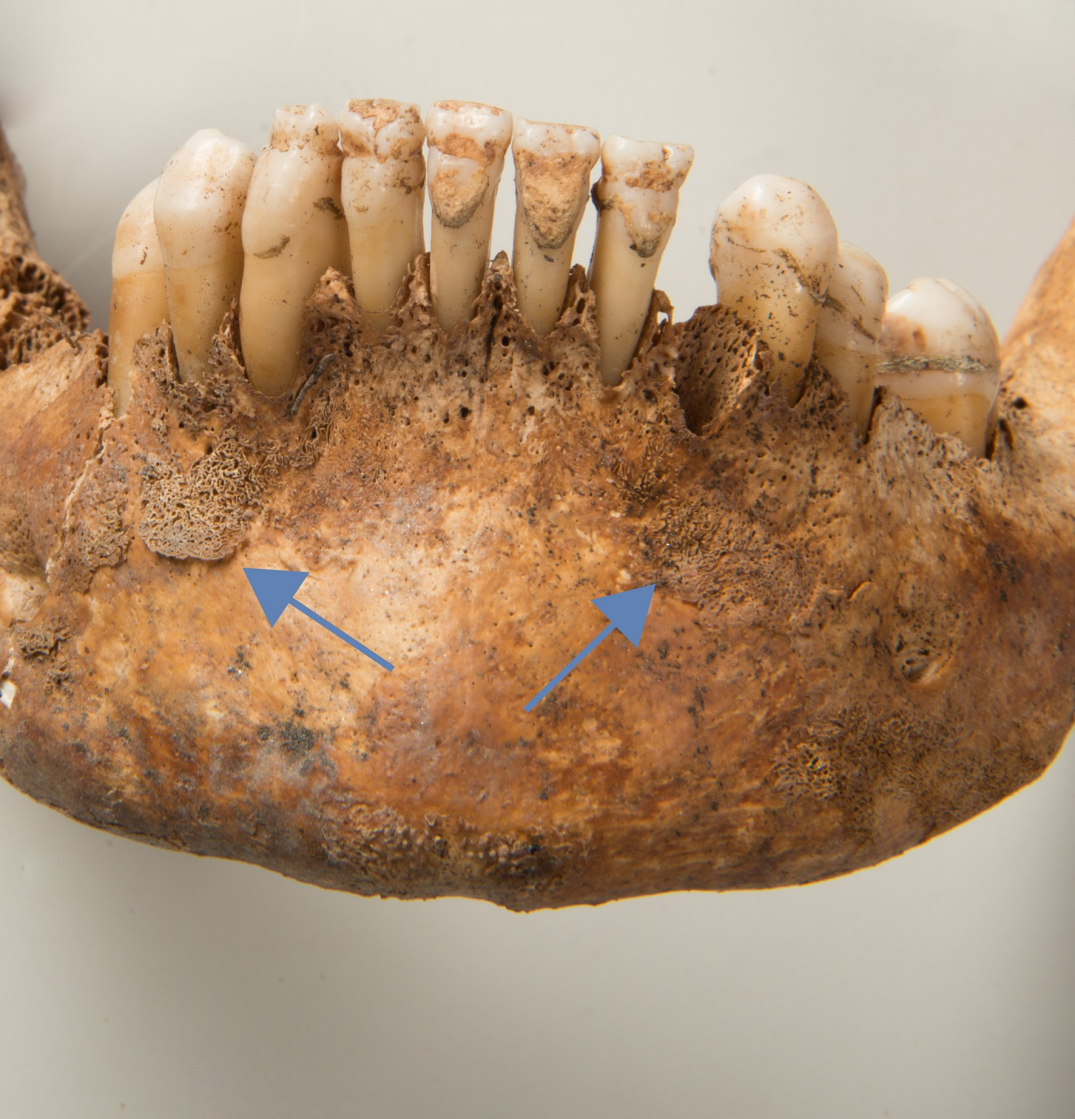
New bone formation on the mandible including the alveolar ridge area (g102).

The alterations were the most frequent in the mandible and the sphenoid (in one case in the pterygoid fossae), followed by the frontal (including orbits), the facial bones (in one case at the infraorbital foramen), the parietal and the temporal. In Eschen, the only reference group for which data was available, only 0.2% of all cranial bones (1/441) were affected, whereas in Cazis/Realta 2.5% (27/1075) exhibited ectocranial new bone formation (P = 0.0012).

The endocranial aspect of the skull was assessable in 101 individuals, six of which exhibited new bone formation and/or vascularisation. The lesions were active in four and healed or partly healed in two individuals (S2 Table). In one case, the frontal was affected, in three cases the occipital and in two cases the entire endocranial surface. The prevalence was not significantly different than in the reference groups (Table 5).

**Table 5:** Prevalence of endocranial lesions in Cazis/Realta and reference groups.

	Individuals			P-value
	N observable	N affected	% affected	
Cazis/Realta	101	6	5.9	
Eschen	46	2	2.2	0.4345
Zweisimmen	36	2	2.8	0.6755

#### Postcranial lesions

Periosteal reactions in long bones were identified in eleven males and four females. Out of 1419 long bones, 41 were affected. In three individuals the long bone lesions were active, in twelve they were partly or fully remodelled (S2 Table). The proportion of affected individuals and long bones, as well as the patterning, was inconspicuous compared to the reference groups. Apart from long bones, the scapula, hand bones, pelvis, sacrum, patella and foot bones were affected. The prevalence in the scapula was especially high compared to Eschen (Table 6).

**Table 6:** Periosteal reactions in postcranial bones in Cazis/Realta and reference groups.

			Clavicula	Humerus	Radius	Ulna	Femur	Tibia	Fibula	Total	Scapula	Sternum	Hand (units)	Pelvis	Sacrum	Patella	Foot (units)
**Males**	Cazis/Realta	N	130.5	133.5	136	136	135	136	136	943	120.5	51	132	129.5	63.5	132.5	130.5
		n	2	2	0	1	4	12	10	31	3	0	1	0	1	0	1
		%	1.5	1.5	0	0.7	3	8.8	7.4	3.3	2.5	0	0.8	0	1.6	0	0.8
	Eschen	N	35.5	38.5	41.5	42	47.5	45.5	46	296.5	25	7.5	44	31	7.5	39	31
		n	0	0	1	1	1	2	1	6	0	0	2	0	0	0	0
		%	0	0	2.4	2.4	2.1	4.4	2.2	2	0	0	4.5	0	0	0	0
	Zweisimmen	N	35.25	38.25	37	36.5	37.5	33	29.5	247							
		n	0	0	0	0	0	4	3	7							
		%	0	0	0	0	0	12.1	10.2	2.8							
**Females**	Cazis/Realta	N	67	69	67	68	69	67.5	68.5	476	59	26.5	64.5	62.5	30.5	64.5	63
		n	0	0	0	0	2	3	5	10	2	0	0	1	0	1	0
		%	0	0	0	0	2.9	4.4	7.3	2.1	3.4	0	0	1.6	0	1.6	0
	Eschen	N	59	59.5	62.5	63.5	55.5	58.5	51.5	410	35	10	50	40	7	41	35
		n	1	0	0	1	3	6	4	15	0	0	0	1	0	0	0
		%	1.7	0	0	1.6	5.4	10.3	7.8	3.7	0	0	0	2.5	0	0	0
	Zweisimmen	N	32.75	30.5	29.25	30	27.5	23.25	20.25	193.5							
		n	0	1	0	1	1	5	1	9							
		%	0	3.3	0	3.3	3.6	21.5	4.9	4.7							
**All**	Cazis/Realta	N	197.5	202.5	203	204	204	203.5	204.5	1419	179.5	77.5	196.5	192	94	197	193.5
		n	2	2	0	1	6	15	15	41	5	0	1	1	1	1	1
		%	1	1	0	0.5	2.9	7.3	7.3	2.9	2.8	0	0.5	0.5	1.1	0.5	0.5
	Eschen	N	94.5	98	104	105.5	103	104	97.5	706.5	60	17.5	94	71	14.5	80	66
		n	1	0	1	2	4	8	5	21	0	0	2	1	0	0	0
		%	1.1	0	1	1.9	3.9	7.7	6.3	3	0	0	2.1	1.4	0	0	0
	Zweisimmen	N	68	68.75	66.25	66.5	65	56.25	49.75	440.5							
		n	0	1	0	1	1	9	4	16							
		%	0	1.5	0	1.5	1.5	16	8	3.6							
	Bern-Grosse Schanze	N								1221							
		n								109							
		%								8.9							
	Bern-Holzwerkhof	N								1728							
		n								146							
		%								8.4							

Blank spaces indicate missing data.

Rib lesions on the visceral surface were identified in 18 individuals and 112 ribs. The lesions were in an active state with only one exception. The total prevalence was significantly higher than in the reference groups (Table 7).

**Table 7:** Prevalence of lesions on the visceral surface of ribs in Cazis/Realta and reference groups.

			Individuals	P-Value*	Ribs	P-Value*
**Males**	Cazis/Realta	N	67		1414	
		n	8		42	
		%	11.9		3.0	
	Eschen	N	21		277	
		n	1	0.6804	2	**0.0360**
		%	4.8		0.7	
	Zweisimmen	N	21		402	
		n	3	0.7195	17	0.2049
		%	14.3		4.2	
**Females**	Cazis/Realta	N	34		727	
		n	10		70	
		%	29.4		9.6	
	Eschen	N	32		417	
		n	2	**0.0235**	11	**<0.0001**
		%	6.3		2.6	
	Zweisimmen	N	16		325	
		n	2	0.2923	6	**<0.0001**
		%	12.5		1.8	
**All**	Cazis/Realta	N	101		2141	
		n	18		112	
		%	17.8		5.2	
	Eschen	N	53		694	
		n	3	**0.0477**	13	**<0.0001**
		%	5.7		1.9	
	Zweisimmen	N	37		727	
		n	5	0.6168	23	**0.0253**
		%	13.5		3.2	
	Bern-Grosse Schanze (incl. children)	N	82		1291	
		n	5	**0.0237**	18	**<0.0001**
		%	6.1		1.4	
	Bern-Holzwerkhof (incl. children)	N	150		1869	
		n	6	**0.0004**	20	**<0.0001**
		%	4.0		1.1	

* Significant results are highlighted with bolded P-values.

We explored whether there was a connection between the presence of rib fractures and reactive periostitis at other locations of the ribs not adjacent to the fractures. Individuals with incompletely healed rib fractures were slightly more often affected by reactive periostitis in ribs (3/23) than those without fractures (6/78), but this was not significant (P = 0.4217).

**Fig 6:**
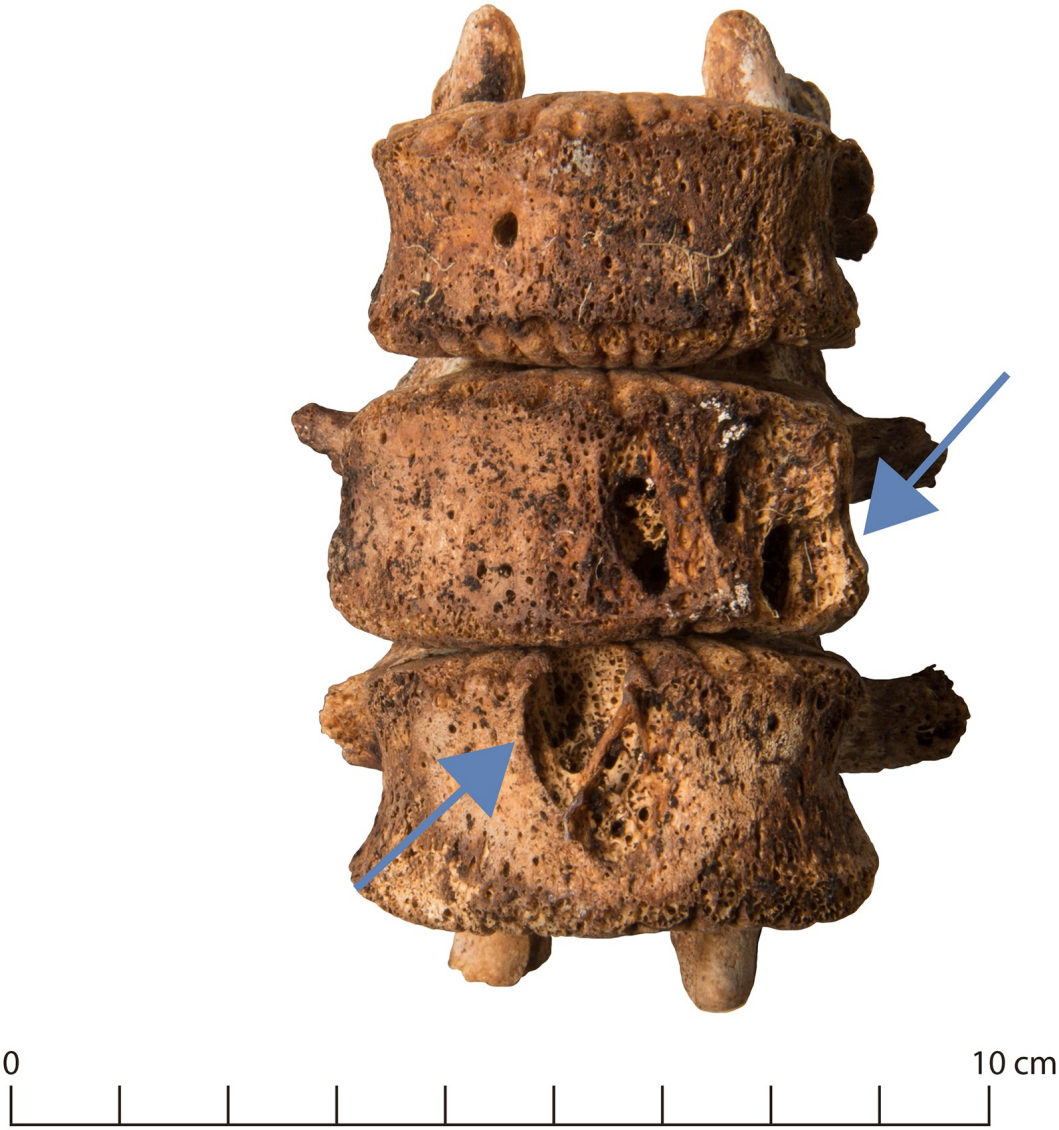
Lytic vertebral lesions (g55).

Lytic vertebral lesions were found in seven male individuals and in 25 vertebrae (S1 Table). In four individuals, the alterations were limited to the endplates and consisted of mostly small, sharply demarcated and purely lytic lesions and diffuse erosion of the endplate, while in three cases they affected the anterior portion of the vertebral body (Fig 6).

The prevalence of lytic vertebral lesions did not differ significantly between Cazis/Realta and the reference groups (Table 8).

**Table 8:** Prevalence of lytic lesions in vertebrae.

			Individuals	P-Value*	Cervical vertebrae	Thoracic vertebrae	Lumbar vertebrae	Total vertebrae	P-Value*
**Males**	Cazis/Realta	N	68		339	720	322	1381	
		n	7		0	15	10	25	
		%	10.3		0	2.1	3.1	1.8	
	Eschen	N	22		76	171	73	320	
		n	2	1.0000	0	2	3	5	0.3915
		%	9.0		0	1.2	4.1	5.3	
	Zweisimmen	N	21		117	179	63	359	
		n	2	1.0000	0	1	1	2	0.0964
		%	9.5		0	0.6	1.6	2.2	
**Females**	Cazis/Realta	N	33		177	348	151	676	
		n	0		0	0	0	0	
		%	0		0	0	0	0	
	Eschen	N	34		153	194	73	420	
		n	3	0.2388	3	0	0	3	0.0560
		%	8.8		2.0	0	0	0.7	
	Zweisimmen	N	17		93	148	69	310	
		n	3	**0.0347**	1	3	4	8	**<0.0001**
		%	17.6		1.1	2.0	5.8	2.6	
**All**	Cazis/Realta	N	101		516	1068	473	2057	
		n	7		0	15	10	25	
		%	6.9		0	1.4	2.1	3.5	
	Eschen	N	56		229	365	146	740	
		n	5	0.7560	3	2	3	8	0.8456
		%	8.9		1.3	0.5	2.1	3.9	
	Zweisimmen	N	38		210	327	132	669	
		n	5	0.3090	1	4	5	10	0.5566
		%	13.2		0.5	1.2	3.8	5.5	

* Significant results are highlighted with bolded P-values.

**Fig 7:**
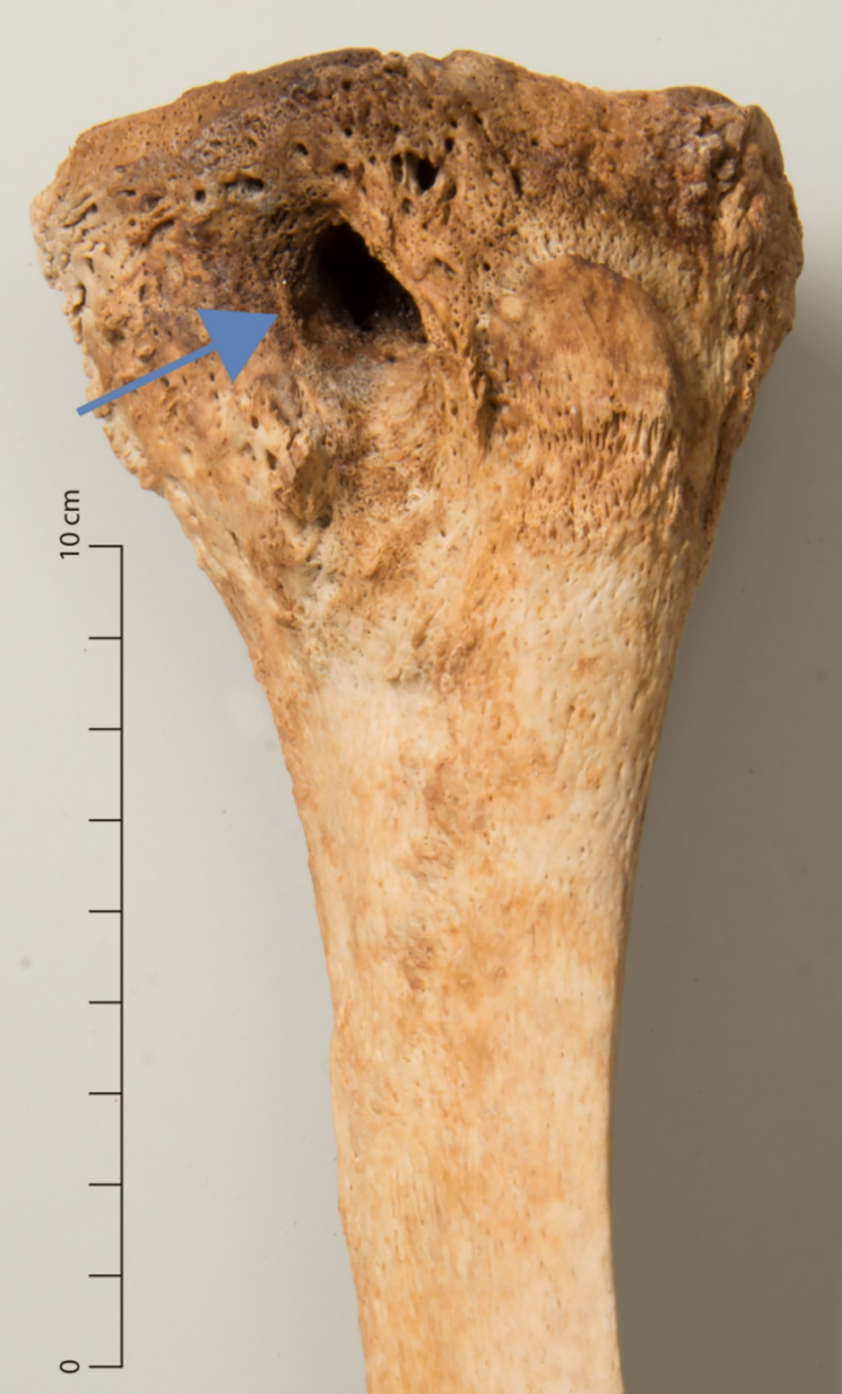
Periarticular lytic lesion in a tibia (g41).

Articular lesions were observed in two individuals. One male (g41) exhibited a periarticular lytic lesion in the proximal left tibia as well as slight proliferative lesions on the surrounding tibial and fibular surfaces (Fig 7). In a female (g67), erosion of the subchondral bone in all articular surfaces of the left knee and some new bone formation around the joint were noted.

#### Other lesions

**Fig 8:**
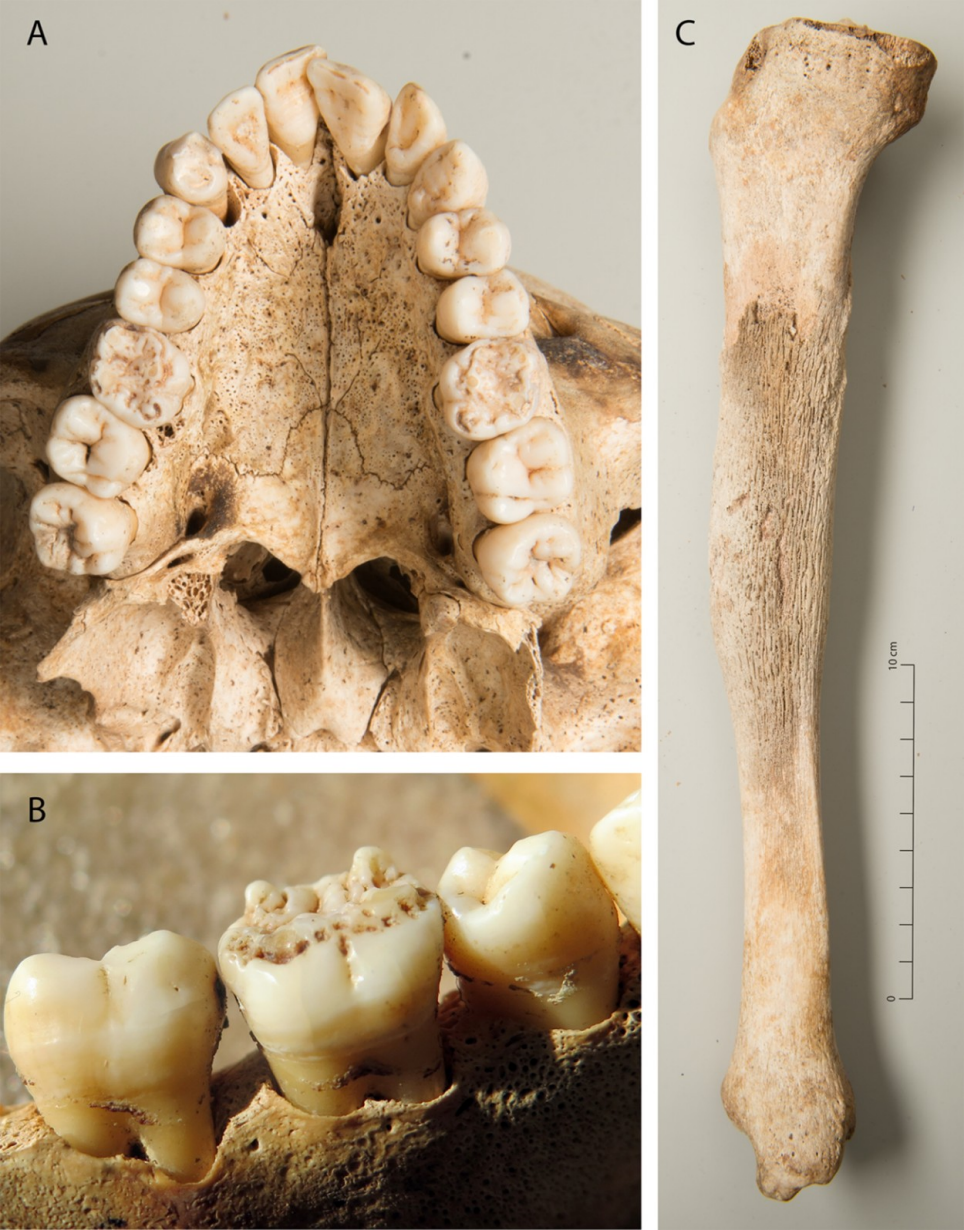
Dental stigmata and long bone lesion (g61). A, B: Mulberry molars, C: Periosteal reaction in the tibia.

In a 20–25 year old male (g61) individual the mandibular molars were tipped inwards and all first molars exhibited hypoplastic defects that characterised them as Mulberry molars [62]. The first maxillary incisors showed slight furrow-like hypoplastic defects around the incisal ridge, consistent with a very mild expression of Hutchinson’s incisors [63]. The palate was high and narrow with an almost triangular shape. A thick layer of newly formed bone (partly remodelled) was found on the right tibia (Fig 8).

### Trauma

#### Cranial trauma

**Fig 9:**
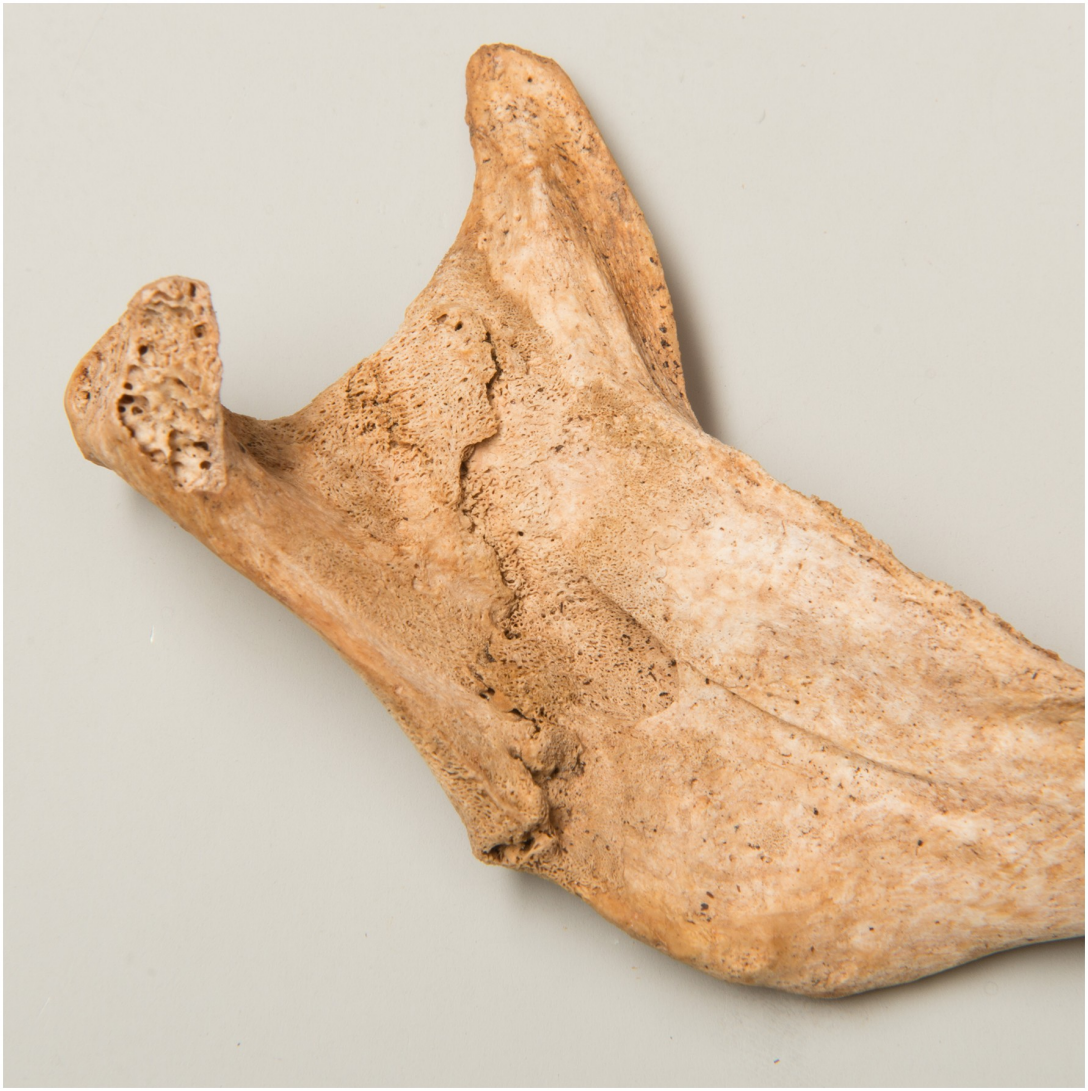
Incompletely healed mandibular fracture (g10).

**Fig 10:**
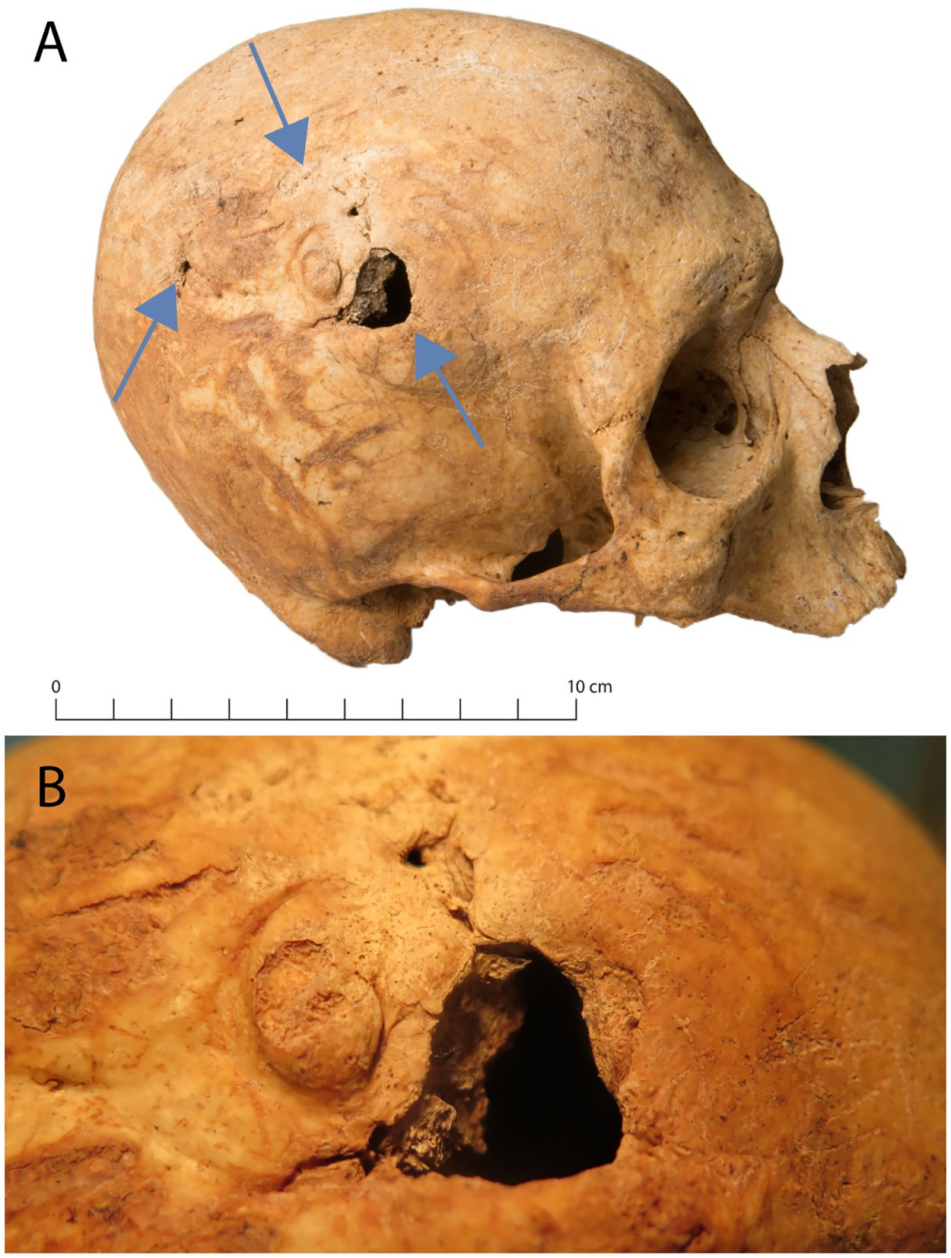
Healed blunt force injury in the parietal (g71). A: overview, B: detail.

Cranial trauma was encountered in four males. In two cases, the mandibular condyle exhibited a completely healed fracture (g29, g73), one of which was associated with mandibular and maxillary dental fractures. In one case, an incompletely healed fracture was found in the ascending ramus (g10, Fig 9). The fourth case (g71) consisted of a healed blunt force injury to the right parietal (Fig 10). A large bone fragment was depressed. At the anterior part the injury penetrated both tables and a bone fragment protruding inwards remained attached to the edge. In Eschen, no cranial traumata were recorded, whereas in Cazis/Realta 3.9% (4/102) of all assessable skulls were affected.

#### Fractures of long bones

Long bone fractures were observed in six females and thirteen males (S3 Table).

**Fig 11:**
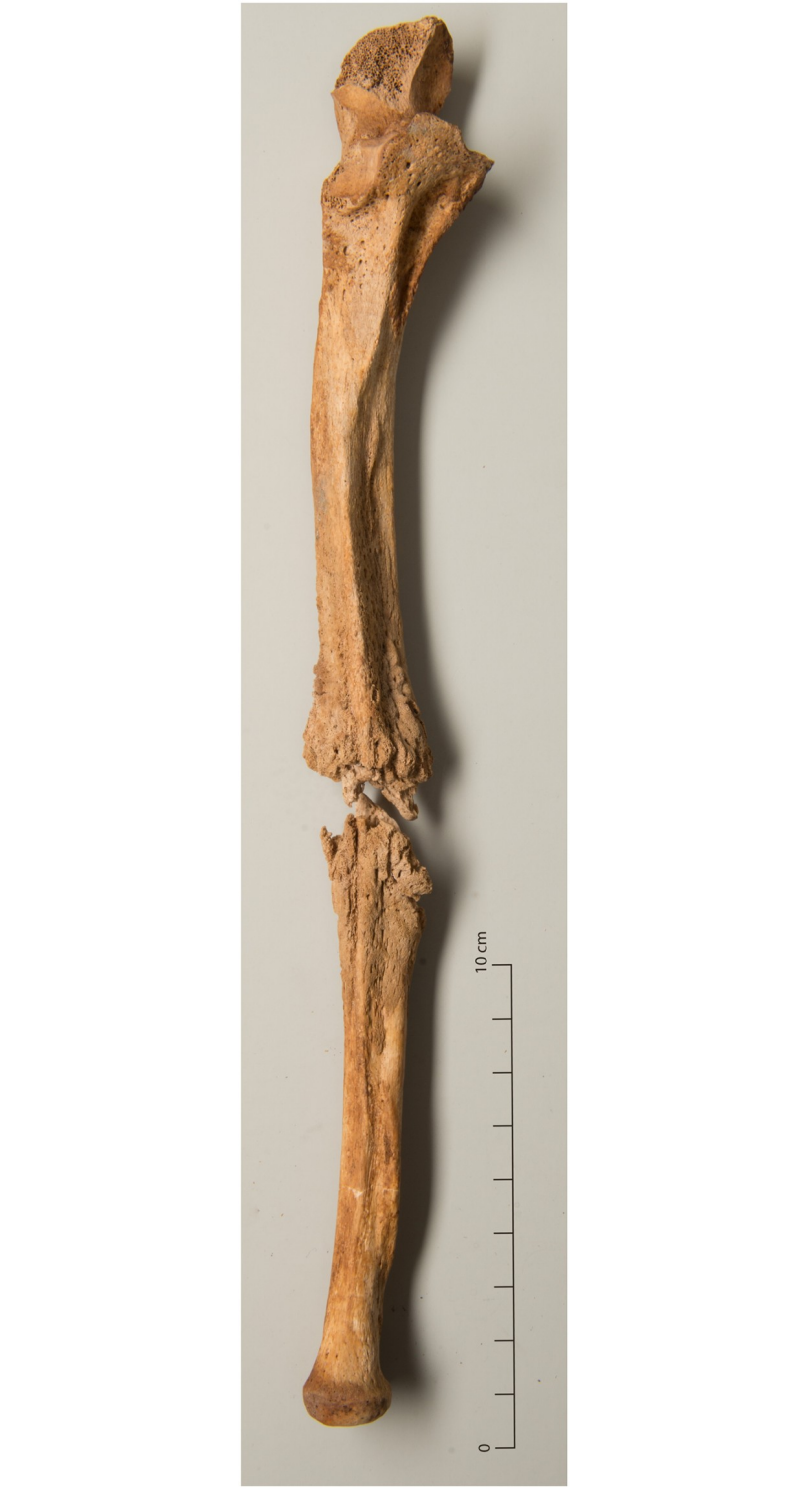
Incompletely healed fracture of the ulna (g10).

**Fig 12:**
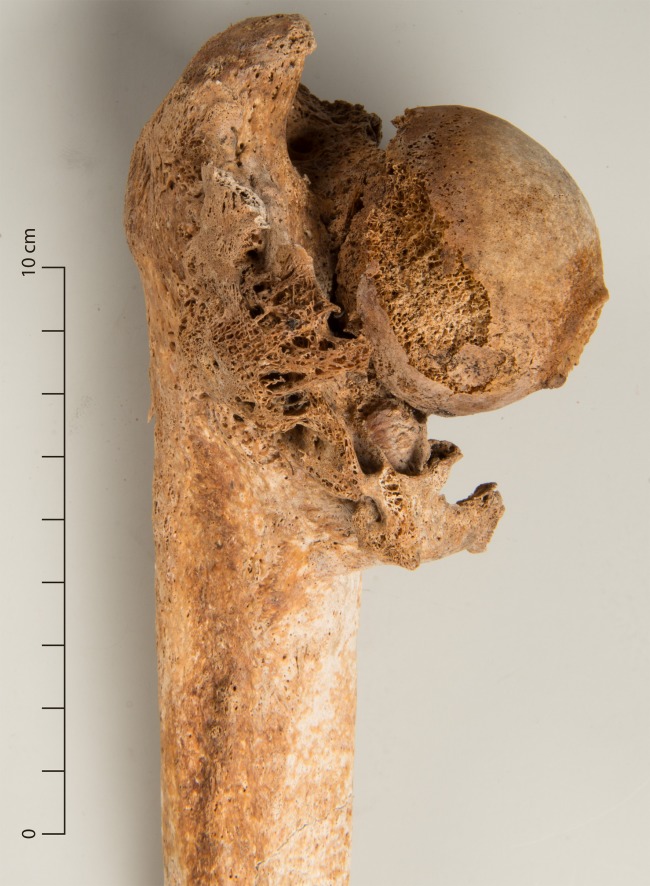
Impacted and incompletely healed proximal femur fracture (g80).

Fractures of the clavicle were found in three females and two males. All, except one, were completely healed, as were the distal radius fractures in two males and two females. Ulnar fractures were found only in males. Two were mid-diaphyseal fractures, one of which was incompletely healed (Fig 11). The others were a healed parry fracture, a fracture of the olecranon and a fracture of the styloid process. In two elderly individuals a fracture of the femoral neck was noted, one of which was healed. The other appeared impacted and was not completely healed (Fig 12). Healed fractures of the tibia and/or fibula were found in four males. The overall long bone fracture rate in Cazis/Realta was significantly higher than in Eschen and Bern-Holzwerkhof (Table 9).

**Table 9:** Fracture rates of long bones (incl. clavicle) in Cazis/Realta and reference groups.

			Individuals	P-Value*	Clavicula	Humerus	Radius	Ulna	Femur	Tibia	Fibula	Total	P-Value*
**Males**	Cazis/Realta	N	68		130.5	133.5	136	136	135	136	136	943	
		n	13		2	0	2	5	1	4	4	18	
		%	19.1		1.5	0	1.5	3.7	0.7	2.9	2.9	1.9	
	Eschen	N	26		35.5	38.5	41.5	42	47.5	45.5	46	296.5	
		n	2	0.2219	0	0	0	1	0	0	1	2	0.1885
		%	7.7		0	0	0	2.4	0	0	2.2	0.7	
	Bern-Grosse Schanze	N			76.5	74	76.5	76	79	72	70.5	524.5	
		n			0	1	2	2	1	1	2	9	0.8427
		%			0	1.4	2.6	2.6	1.3	1.4	2.8	1.7	
	Bern-Holzwerkhof	N			90.5	90.5	89	86	90	70.5	60.5	577	
		n			0	0	1	0	0	0	0	1	**0.0017**
		%			0	0	1.1	0	0	0	0	0.2	
	Zweisimmen	N	21		35.25	38.25	37	36.5	37.5	33	29.5	247	
		n	2	0.5058	1	0	1	0	0	0	0	2	0.4011
		%	9.5		2.8	0	2.7	0	0	0	0	0.8	
**Females**	Cazis/Realta	N	35		67	69	67	68	69	67.5	68.5	476	
		n	6		3	0	2	0	1	0	0	6	
		%	17.1		4.5	0	3.0	0	1.4	0	0	1.3	
	Eschen	N	36		59	59.5	62.5	63.5	55.5	58.5	51.5	410	
		n	1	0.0553	0	0	0	0	1	0	0	1	0.1308
		%	2.8		0	0	0	0	1.8	0	0	0.4	
	Bern-Grosse Schanze	N			59	58.5	63	60.5	64	61	57.5	423.5	
		n			1	0	1	0	0	0	0	2	0.2935
		%			5.1	0	1.6	0	0	0	0	0.5	
	Bern-Holzwerkhof	N			116	120	124	121	95.5	57	51.5	685	
		n			3	0	0	0	0	0	0	3	0.1721
		%			2.6	0	0	0	0	0	0	0.4	
	Zweisimmen	N	17		32.75	30.5	29.25	30	27.5	23.25	20.25	193.5	
		n	0	0.1605	0	0	0	0	0	0	0	0	0.1890
		%	0		0	0	0	0	0	0	0	0	
**All**	Cazis/Realta	N	103		197.5	202.5	203	204	204	203.5	204.5	1419	
		n	19		5	0	4	5	2	4	4	24	
		%	18.1		2.5	0	2.0	2.5	1	2	2	1.7	
	Eschen	N	62		94.5	98	104	105.5	103	104	97.5	706.5	
		n	3	**0.0166**	0	0	0	1	1	0	1	3	**0.0128**
		%	4.8		0	0	0	0.9	1	0	1	0.4	
	Bern-Grosse Schanze	N			135.5	132.5	139.5	136.5	143	133	128	948	
		n			1	1	3	2	1	1	2	11	0.3851
		%			0.7	0.8	2.2	1.5	0.7	0.8	1.6	1.2	
	Bern-Holzwerkhof	N			206.5	210.5	213	207	185.5	127.5	112	1262	
		n			5	0	1	0	0	0	0	6	**0.0028**
		%			2.4	0	0.5	0	0	0	0	0.5	
	Zweisimmen	N	38		68	68.75	66.25	66.5	65	56.25	49.75	440.5	
		n	2	0.0625	1	0	1	0	0	0	0	2	0.0616
		%	5.3		1.5	0	1.5	0	0	0	0	0.5	

* Significant results are highlighted with bolded P-values.

Blank spaces indicate missing data.

**Fig 13:**
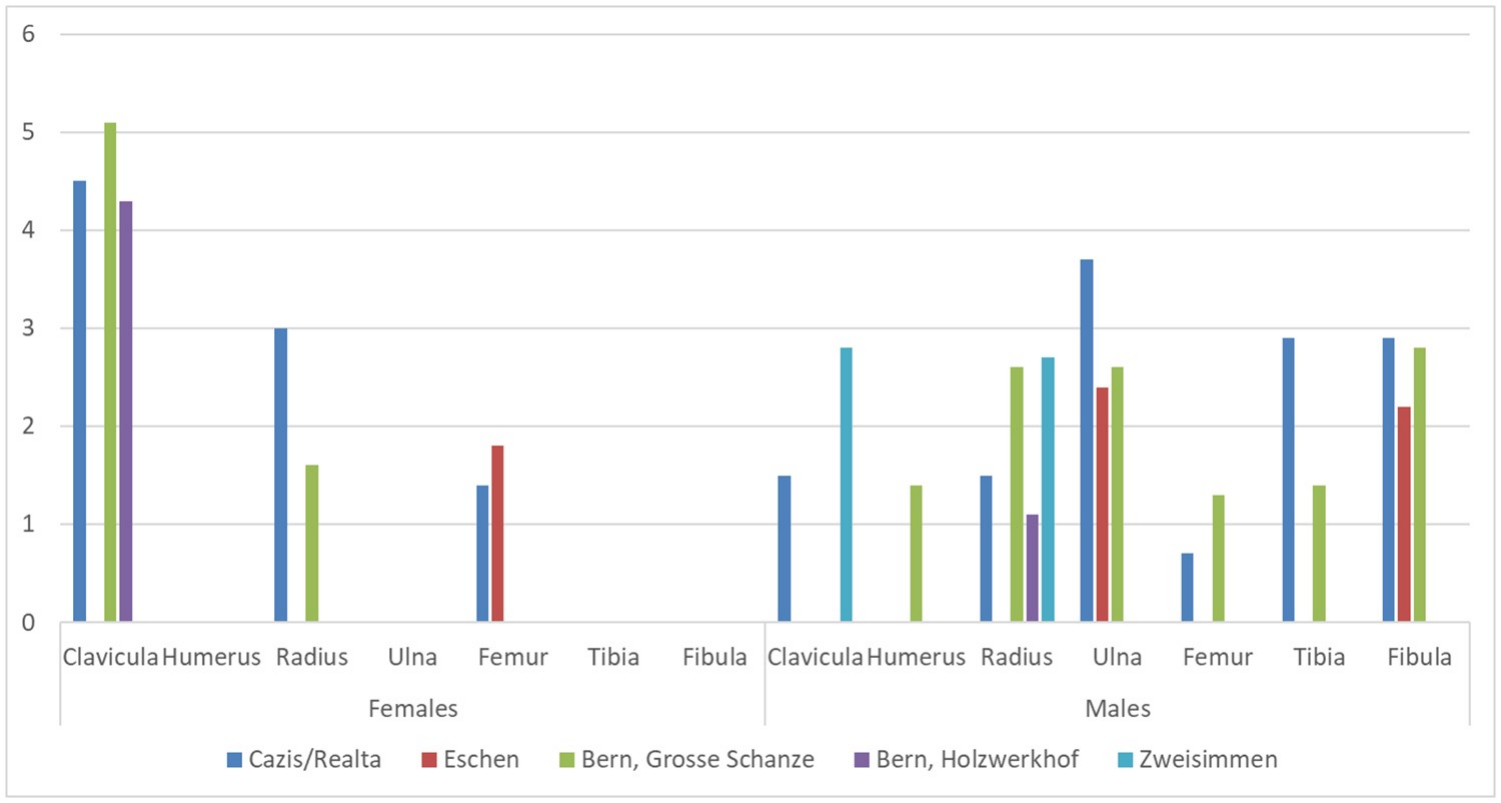
Fracture rates (%) of individual long bones in Cazis/Realta and reference groups.

A distinct sex-specific fracture pattern was observed. In females, fractures were limited to the clavicle, the radius and the proximal femur, whereas in males all long bones, except the humerus, were affected, with the highest fracture rates found in the ulna, tibia and fibula. Similar general patterns were also seen in the reference groups. The congruency between Cazis/Realta and Bern-Grosse Schanze was especially noteworthy (Fig 13).

#### Fractures of other postcranial bones

**Fig 14:**
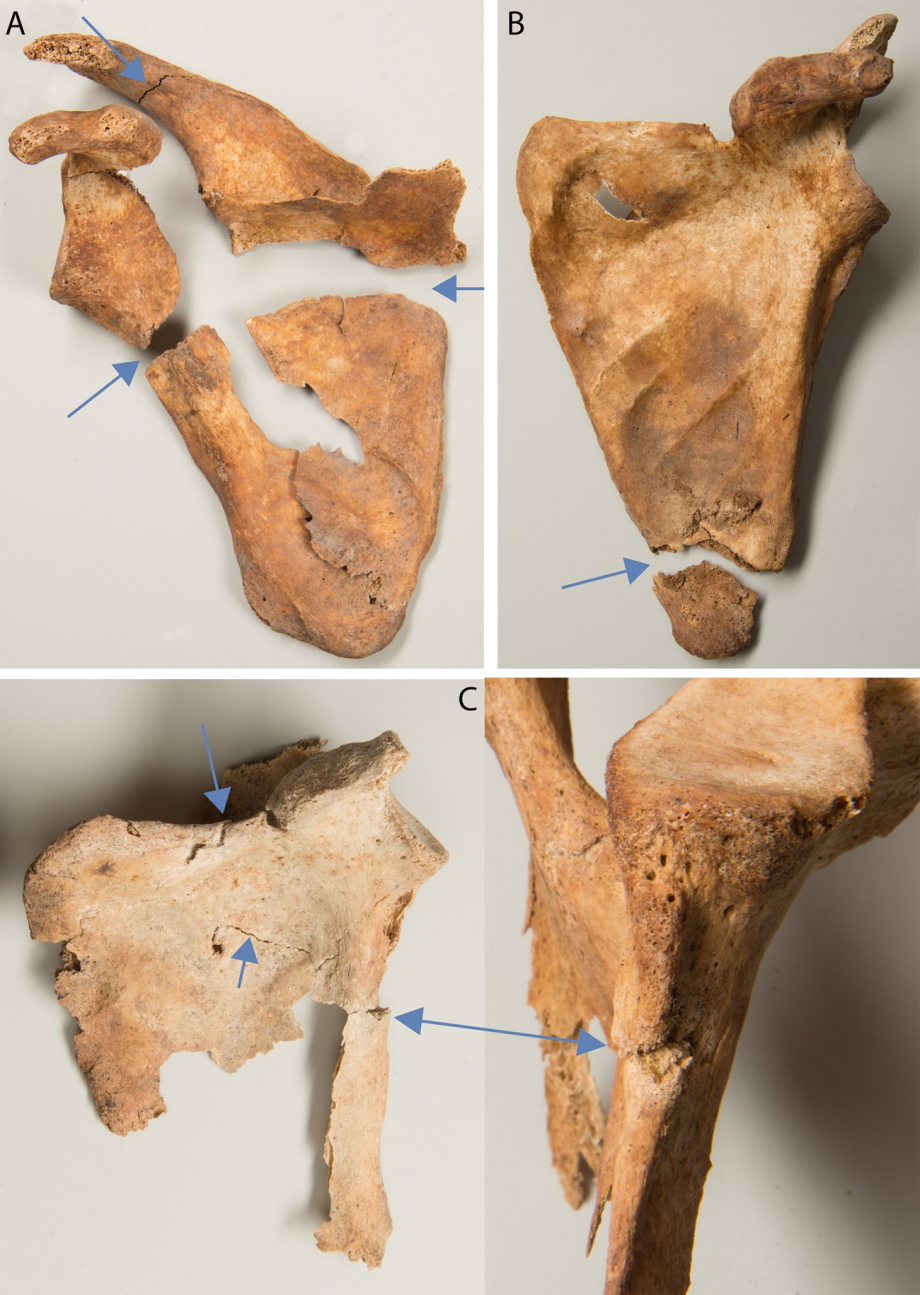
Scapular fractures. A: perimortem fracture (g3), B: complete fracture at the inferior angle (g71), C: incomplete fractures at the lateral and superior margin (g51).

Traumata of other postcranial bones were identified in 37 males and 16 females (S3 Table). Vertebral fractures affected eight males and two females. Two males exhibited a fracture through the spinous process («clay shoveller fracture») of the first thoracic vertebra. In five individuals, crush fractures of the vertebral body were associated with pathological bone loss. Compression fractures were found in three males, in two of which they were incompletely healed. Scapular fractures were found in three females and five males. In two cases, they were bilateral. A complete fracture of the neck that separated the glenoid from the body and showed no signs of healing was found in the left scapula of a male (Fig 14A). In one male a complete fracture through the inferior angle was seen (Fig 14B). The remaining fractures were all incomplete fractures in the form of clefts or fissures (Fig 14C). They affected the medial, lateral and superior margin, the acromion, the body, and the spine of scapula in different combinations. All were incompletely healed.

**Fig 15:**
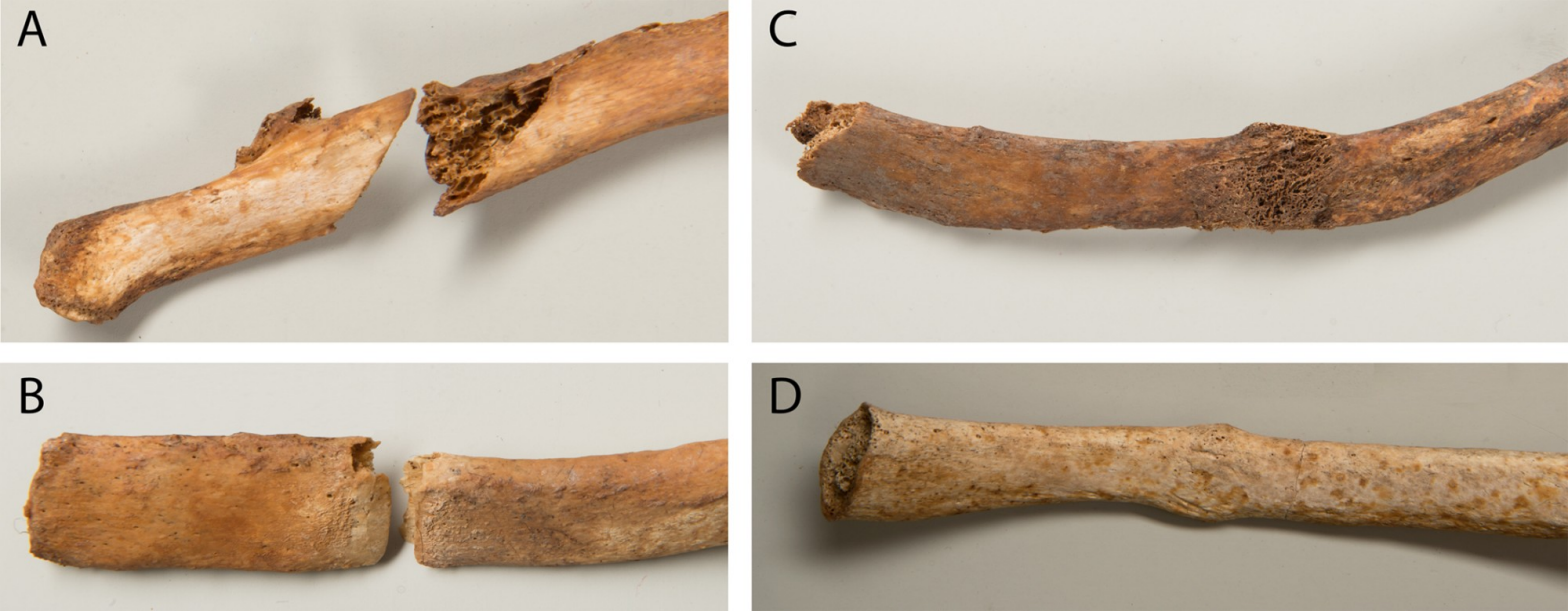
Rib fractures in different stages of healing. A: stage 1, B: stage 2, C: stage 3, D: stage 4.

Rib fractures (Fig 15) were found in 14 females and 31 males (S4 Table). Out of the total 380 rib fractures, eleven were perimortem (stage 1), 138 were in a very early stage of healing (stage 2), 40 exhibited a calcified callus (stage 3) and 191 were remodelled (stage 4).

Two females and five males exhibited fractures of hand bones (hamate, capitate, metacarpals and phalanges). All except a spiral fracture of a metacarpal were completely healed. Pelvic fractures were recorded in two females and one male. In the females, the pubic bone showed a fracture in an early stage of healing. In the male (g98) the acetabulum and both pubic rami were fractured. Secondary to this trauma, a *protrusio acetabuli* (medial displacement of the acetabulum and femoral head) with severe degenerative changes had developed.

A healed incomplete patellar fracture was identified in one male. Four males and two females exhibited fractures in bones of the feet, four of which were articular impression fractures of the talus. Another was an incomplete intraarticular fracture in the calcaneus. The proximal phalanx was fractured in one female, whereas in one male the distal end of the first metatarsal was atrophied and the joint as well as the phalanges were missing, suggesting a healed amputation of the toe.

Reference data based on bone counts was only available for Eschen, except for rib fractures for which data was recorded at other sites as well. The most striking difference was found in the prevalence of rib fractures that was significantly higher in Cazis/Realta than in all reference groups. Furthermore, high fracture rates were recorded for bones that did not exhibit any fractures at all in Eschen, most notably the vertebrae and scapulae (Table 10).

**Table 10:** Fracture rates of other postcranial bones in Cazis/Realta and reference groups.

			Ribs (individuals)	P-Value*	Ribs (bone count)	P-Value*	Spine (units)	Scapula	Sternum	Hand (units)	Pelvis	Sacrum	Patella	Foot (units)
**Males**	Cazis/Realta	N	67		1414		185	121	51	132	129.5	64	133	131
		n	31		191		10	7	0	5	1	0	1	4
		%	46.3		13.5		5.4	5.8	0	3.8	0.8	0	0.8	3.1
	Eschen	N	21		277		43	25	7.5	44	31	7.5	39	31
		n	3	**0.0100**	7	**<0.0001**	0	0	0	1	0	0	0	0
		%	14.3		2.5		0	0	0	2.3	0	0	0	0
**Females**	Cazis/Realta	N	34		727		90.5	59	26.5	64.5	62.5	30.5	64.5	63
		n	14		87		2	3	0	2	2	0	0	3
		%	41.2		12		2.2	5.1	0	3.1	3.2	0	0	4.8
	Eschen	N	32		417		59	35	10	50	40	7	41	35
		n	2	**0.0012**	3	**<0.0001**	0	0	0	0	0	0	0	0
		%	6.3		0.7		0	0	0	0	0	0	0	0
**All**	Cazis/Realta	N	101		2141		275.5	180	77.5	196.5	192	94.5	197.5	194
		n	45		278		12	10	0	7	3	0	1	7
		%	44.6		13		4.3	5.6	0	3.6	1.6	0	0.5	3.6
	Eschen	N	53		694		102	60	17.5	94	71	14.5	80	66
		n	5	**<0.0001**	10	**<0.0001**	0	0	0	1	0	0	0	0
		%	9.4		1.4		0	0	0	1.1	0	0	0	0
	Bern-Grosse Schanze	N			1291									
		n			29	**<0.0001**								
		%			2.2									
	Bern-Holzwerkhof	N			1869									
		n			35	**<0.0001**								
		%			1.9									
	Zweisimmen	N	37		727									
		n	4	**0.0006**	6	**<0.0001**								
		%	10.8		0.8									

* Significant results are highlighted with bolded P-values.

Blank spaces indicate missing data.

#### Stages of healing

**Fig 16:**
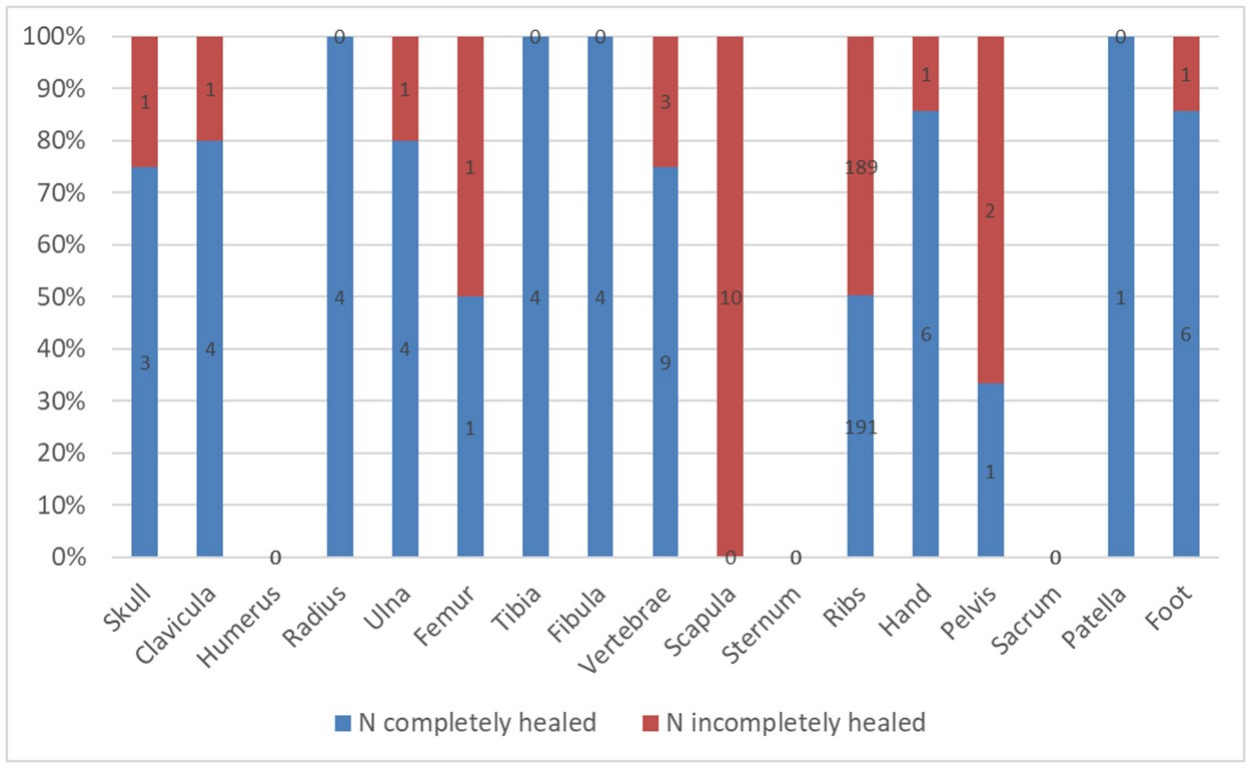
Proportion of completely and incompletely healed fractures in different bones.

The proportions of healed and incompletely healed fractures differed depending on the affected part of the skeleton. Most cranial and long bone fractures were completely healed, but in other postcranial bones, over 60% of the fractures were not. The proportion of incompletely healed fractures was particularly large in the scapula and the ribs (Fig 16).

The individuals with incompletely healed fractures comprised 12 females and 20 males. In 16 of these individuals, the incompletely healed fractures were restricted to ribs, in another five only the ribs and the scapula were affected (Table 11).

**Table 11:** Patterning of incompletely healed traumata.

Affected bone(s)	N affected individuals		
	Females	Males	Total
Ribs	6	10	16
Ribs and scapula	2	3	5
Ribs and long bone	1	1	2
Scapula	0	1	1
Pelvis	1	0	1
Ribs, scapula and pelvis	1	0	1
Mandible, ribs and long bone	0	1	1
Ribs, scapula and vertebrae	0	1	1
Ribs and vertebrae	0	1	1
Vertebrae	0	1	1
Ribs and hand	0	1	1
Ribs and foot	1	0	1
Total	12	20	32

#### The influence of pathological bone loss

In six females and five males, the bones were remarkably light, thin-walled and brittle, suggesting considerable bone loss. To estimate the extent to which these individuals influenced the overall picture, separate postcranial fracture rates for the subgroups with and without obvious bone loss were calculated (Table 12). As expected, the fracture rates in the subgroup with bone loss were higher, but they did not greatly influence the overall fracture rates of the whole sample.

**Table 12:** Postcranial fracture rates in individuals with and without obvious bone loss.

Fractures	Sub-group	N observable bones/units	N affected	% affected
Long bones	Bone loss	138	4	2.9
	No bone loss	1281	20	1.6
	All	1419	24	1.7
Other postcranial bones	Bone loss	162	23	14.2
	No bone loss	1419	85	6
	All	1581	108	6.8
Ribs	Bone loss	216	71	32.9
	No bone loss	1925	207	10.8
	All	2141	278	13
Incompletely healed postcranial fractures	Bone loss	27	15	55.6
	No bone loss	102	53	52
	All	129	68	52.7

### Skeletal deformities

**Fig 17:**
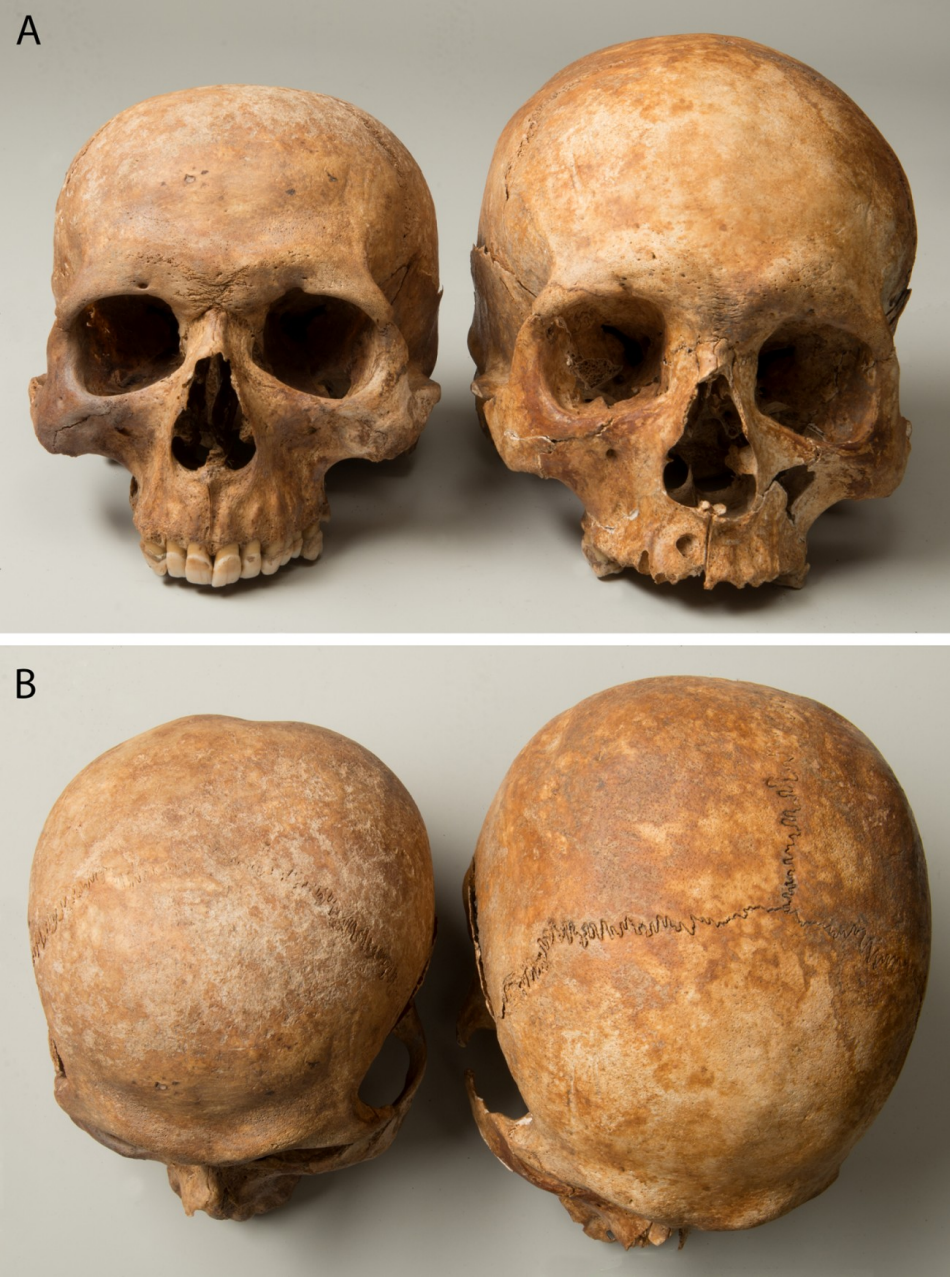
Skull with exceptionally small vault (g69) in comparison to an unremarkable skull. A: view from the front, B: view from above.

Three cases of craniosynostosis (premature closure of cranial sutures) were identified. One individual exhibited moderate plagiocephaly (diagonal asymmetry of the skull). Another mild case of plagiocephaly was seen in a male with other malformations (g20, see below). In one case the left temporal suture was completely fused without cranial deformation. The cranial vault of one male was strikingly small with a circumference of 44 cm (g69). The facial bones appeared large in comparison to the vault, and the frontal was receding (Fig 17).

Scoliosis, an abnormal lateral curvature of the spine, was identified in four male individuals. In two cases the expression was mild in two it was more pronounced.

**Fig 18:**
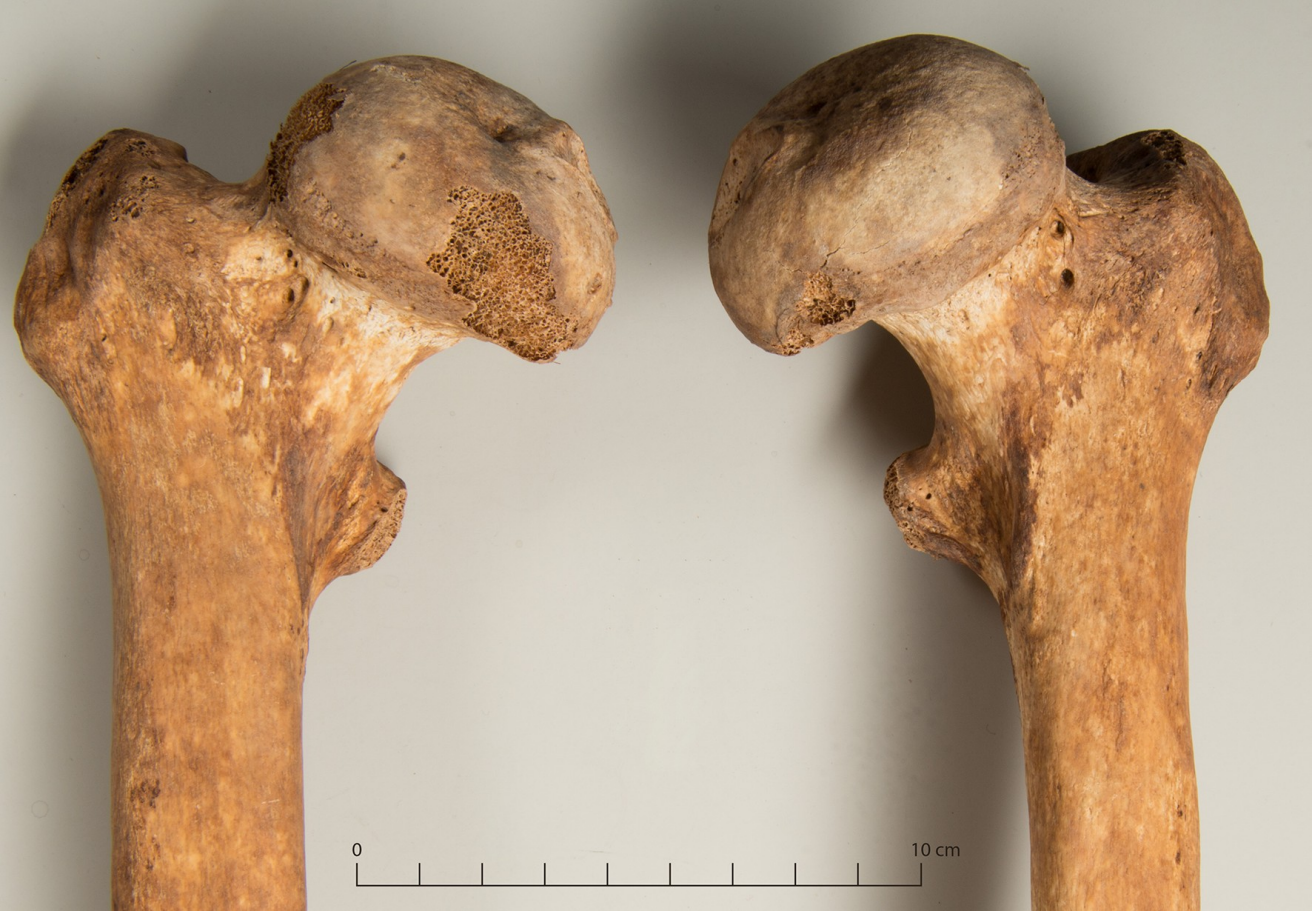
Bilateral dysplasia of the femoral head (g88).

A malformation of the hip joint was recorded in four male individuals. In one individual the condition was unilateral, in three it was bilateral. In all cases, the femoral head was mushroom-shaped and flattened, with an accordingly shallow and wide acetabulum (Fig 18). An abnormal angle of femoral anteversion was found unilaterally in one case and bilaterally in another with secondary degenerative changes of the knee joint.

**Fig 19:**
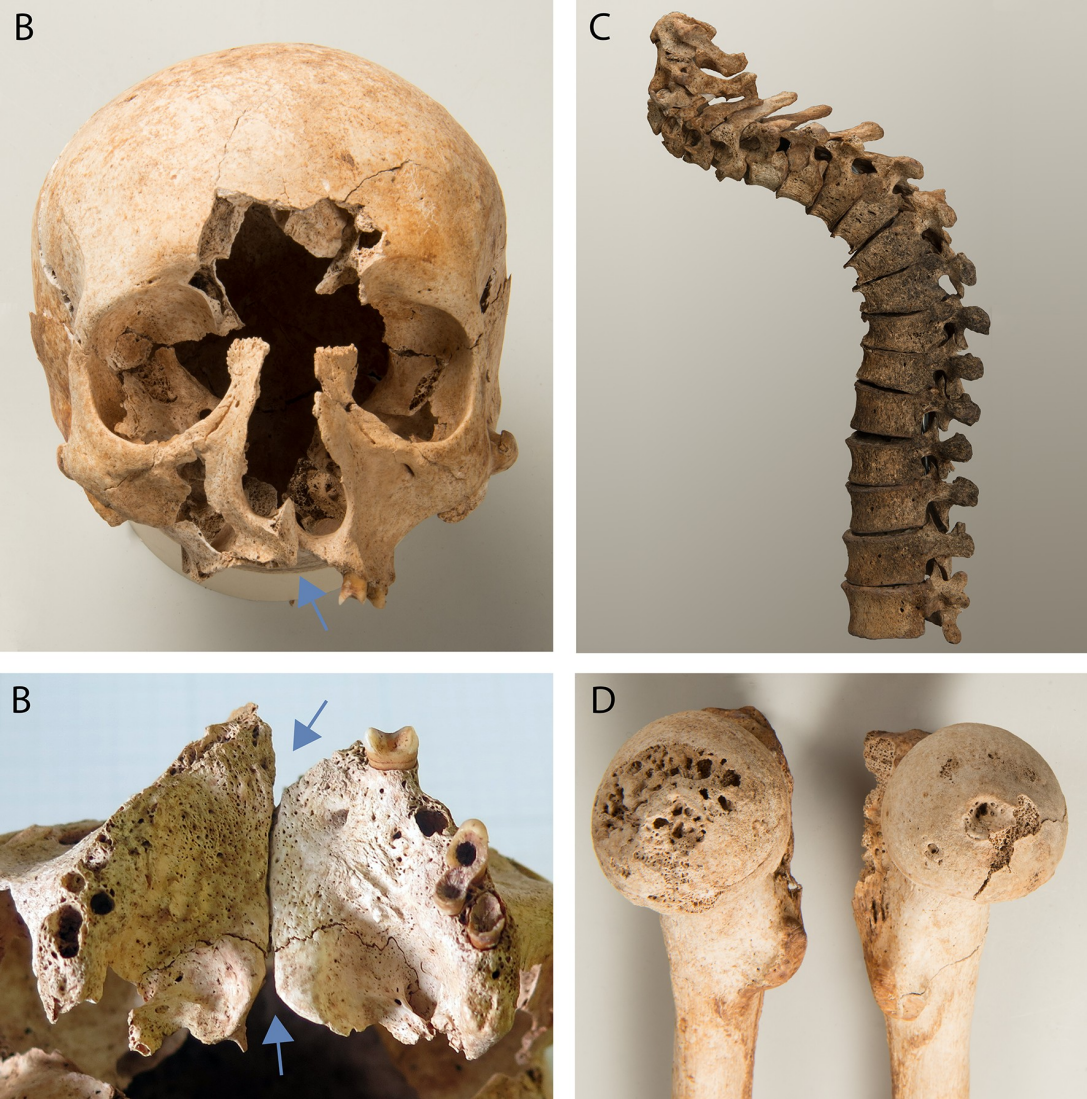
Individual with a combination of malformations (g20). A, B: maxillary cleft and hypoplastic zygomatic bones and maxilla, C: pronounced kyphosis in the thoracic and lordosis in the cervical spine, D: unilateral disintegration of the bone surface in the femoral head.

A male (g20) exhibited a combination of different malformations (Fig 19). The skull was characterised by a cleft in the maxilla that extended from the left bony palate into the piriform aperture and affected the spina nasalis posterior as well. The maxilla, the mandible and both zygomatic bones were hypoplastic. Postmortem breakage around the glabella hindered a proper assessment, but the preserved parts suggested an unusually wide and flat nasal bridge. Furthermore, a slight plagiocephalic deformation indicative of craniosynostosis was observed. The thoracic vertebrae 2–6 were wedge-shaped, leading to a pronounced kyphosis. Corresponding changes in the facet joints of the cervical vertebrae suggested abnormal lordosis in this segment. The vertebral end plates appeared irregular overall, and additionally Schmorl’s nodes (bone defects of the vertebral body due to herniation of the intervertebral disk’s nucleus pulposus) were identified in the thoracic vertebrae 5–12. The contours of many joints appeared to be flattened and less pronounced than normal. Both proximal humeral joints exhibited smooth, groove-like pits at the anterior portion. The femoral heads were flat and mushroom-shaped, and their angle corresponded to a *coxa valga*. A disintegration of the bone surface was seen in the right femoral head as well as in the iliosacral joints and the pubic symphysis. Smooth grooves, disintegration and caverns around the margins were also noted in several joints of the hands and feet.

## Discussion

### Demographic composition

While the unbalanced sex ratio did not correspond to findings in regular cemetery samples with approximately equal proportions of males and females [58,59], it was consistent with historic data. Between 1855 and 1918, a total of 1414 admissions to the institution were recorded, roughly 75% of which were males and 25% females [4]. Considerably more men than women were detained at such facilities in other cantons as well [1,3]. Unbalanced sex ratios with a predominance of males were also found in the cemeteries of the poorhouse in Riggisberg [32,33] and the psychiatric hospital in Hall [31] and therefore appear to be a common feature of skeletal assemblages from such backgrounds.

The age distribution in the skeletal sample differed from 19^th^ century death register data in that there were no children and most adult deaths occurred between 40 and 60 years of age rather than above 60 years (Fig 3, Table 3). This was consistent with the information from the institution’s archival records, although it must be noted that the ages at death of only 54 persons were found in them. The absence of children suggests that they were admitted to separate institutions like orphanages and children’s homes [4]. Two juvenile individuals in the sample indicate that adolescents were occasionally detained in Cazis/Realta though. There are several possible reasons for the underrepresentation of elderly individuals. Correctional measures, such as forced labour, were probably aimed at younger individuals, and the elderly may have been allocated to other institutions such as poorhouses instead. Furthermore, some reasons for detention like alcoholism and hereditary conditions, as well as the living conditions at the institution (see below) may have lowered the life expectancy of detainees.

### Ectocranial changes: Evidence of scurvy

Ectocranial new bone formation (Table 4) was suggestive of scurvy, the result of a deficiency in vitamin C. This vitamin is not synthesized by the human body and must be obtained from dietary sources such as fresh vegetables and fruits [64,65]. Skeletal changes only occur after vitamin C is reintroduced after a period of deficiency [65,66]. In adults, porosity and new bone formation in the greater wing of the sphenoid, the maxilla (posterior and alveolar process) and the mandible (medial coronoid process and alveolar process) are considered to be nearly pathognomic for scurvy [24,66,67,68,69]. Further locations that can be involved include the infraorbital foramen, the lesser wing of the sphenoid, long bones, the endocranial surface of the parietal and the area of the mylohyoid line of the mandible [24,69]. New bone formation in the orbits [67] and the presence of palatine tori or abnormal porosity of the hard palate [70,71] have also been considered suggestive of scurvy in adults.

Based on the criteria above, skeletal changes in at least two locations suggestive of scurvy were identified in five individuals from Cazis/Realta, though in one case the alterations may be linked to congenital syphilis (g61, see below). In the remaining four cases (g47, g76, g90, g102), one of which exhibited partly remodelled lesions, scurvy is the most plausible explanation (Figs 4 and 5). Individuals with lesions in only one location were not diagnosed with scurvy. The comparison with Eschen, where no evidence of scurvy was found, suggests that this deficiency disease was unusually common among the detainees. Scurvy is known to occur in populations with poor nutrition even in present times. The affected groups include the disadvantaged, alcoholics and psychiatric patients, the isolated elderly as well as institutionalized or chronically ill individuals [72]. It cannot be ascertained though whether scurvy was a direct result of detention or of other risk factors mentioned above which would have been faced by many of the inmates.

### Infectious disease

#### A possible case of late congenital syphilis

The combination of probable Hutchinson’s incisors with mulberry molars in a male (g61, Fig 8) is suggestive of late congenital syphilis. Hutchinson’s incisors and Moon’s molars are considered to be pathognomic stigmata of this disease [62], but mulberry molars can also have other causes [73,74]. The transmission of *Treponema pallidum* to the child takes place in utero or during birth from a mother suffering from venereal syphilis [62,73,75]. While the early form of congenital syphilis produces symptoms before the age of two years, the late form can be dormant for many years and lead to skeletal lesions in late childhood or early adult age [52]. When postcranial alterations occur, they mostly affect the tibia, radius and ulna, whereby a saber-shin-like thickening of the tibia—like the partly remodelled lesion in this individual—is typical [52] and further supports the diagnosis. Syphilis was common in Switzerland in the 19^th^ and early 20^th^ centuries before the discovery of penicillin and was considered a disease of prostitutes [76]. In the living patient, the so-called Hutchinson’s triad is pathognomic. It consists of interstitial keratitis, Hutchinson incisors, mulberry molars, and eighth nerve deafness [52]. The affected individual may therefore have been deaf which could have been a reason for being admitted to the institution.

#### Tuberculosis and other pulmonary disease

If a tuberculosis infection is established in the skeleton, it leads to the formation of sharply circumscribed tubercles, followed by gradual destruction and finally the collapse of the skeletal elements [77,78]. The spine is most commonly affected [78] and the lesions are generally in the anterior part of the vertebral body [52,55,77]. One case was diagnosed as a vertebral hemangioma and therefore excluded from the discussion of infectious disease, but in the other two individuals with anterior lytic lesions (g55, g96) a diagnosis of tuberculosis seems plausible (Fig 6). The aetiology of the remaining small lytic lesions in vertebral endplates is difficult to narrow down. They may be an early manifestation of tuberculous spondylodiscitis [79], but other aetiologies can by no means be ruled out. The prevalence of lytic vertebral lesions was unremarkable compared to the reference groups (Table 8).

Besides the spine, the hip and knee joint are the most common locations of skeletal tuberculosis [78]. The periarticular lytic lesion in a tibia (g41, Fig 7) was consistent with an osseous focus that led to a tuberculous abscess [80]. A possible differential diagnosis is osteomyelitis [54,73], however, the lack of significant new bone formation makes this seem unlikely. The erosion of the subchondral bone in all articular surfaces of the knee in one individual (g67) was indicative of tuberculous arthritis, in which granulation tissue gradually erodes the joint cartilage and then the bone beneath it [78]. Differential diagnoses include septic arthritis or some forms of erosive arthropathies, though their patterning does not match the findings in this individual [81].

Proliferative and lytic lesions in ribs, that were active in all but one individual, were found in a significantly higher prevalence than in the reference groups (Table 7). New bone formation on the visceral rib surface can occur in tuberculosis, non-tuberculous pulmonary disease such as pneumonia and bronchitis, cardiac disease and cancer, with tuberculosis leading to lesions far more frequently than the other causative illnesses [82,83,84,85,86]. The pain associated with rib fractures can lead to a decrease in respiratory movement and ventilation of the lungs and thus to pneumonia. However, individuals with incompletely healed rib fractures did not have significantly more rib lesions than those without. Furthermore, changes in the ribs are relatively rare in non-tuberculous pneumonia [73]. Therefore, tuberculosis was most likely the main cause of reactive periostitis in ribs in this group.

New bone formation on scapulae that was found frequently in this group has rarely been mentioned in the palaeopathological literature, though it has been found to be associated with tuberculosis [83,87]. Vascular lesions and new bone formation on the endocranial surface are thought to be mainly caused by pulmonary disease, especially tuberculosis [54,78,88,89,90,91], but the typical tuberculous skull lesions are lytic and perforate both tables [92]. Because the aetiology of endocranial lesions is multifactorial, a certain diagnosis is rarely possible [73,93]. In Cazis/Realta, endocranial lesions, that were active in four out of six cases, were found in average prevalence (Table 5). Altogether, they can most likely be interpreted as another possible indication of tuberculosis.

When lesions occur at multiple sites in the skeleton, this is indicative of a systemic infection, while isolated lesions can result from various factors [94]. The lesions that are generally thought to be associated with skeletal tuberculosis (ribs, scapula, endocranial surface, joints) occurred as isolated alterations but were also found in various combinations (S1 and S2 Tables).

While periosteal reactions in long bones can reflect stimulation of the periosteum by inflammation, nonspecific infections, trauma, strains and lacerations, metabolic or neoplastic conditions, varicose veins and venous stasis oedema [54,73,95], an infectious aetiology seems more likely when they occur together with other lesions suggestive of infection. Altogether, both the prevalence and the patterning of the mostly healed periosteal reactions in long bones corresponded to the findings in reference groups (Table 6).

In summary, there was abundant evidence of tuberculosis in the assemblage. Characteristic tuberculous lesions and combinations of suggestive lesions justified a diagnosis of tuberculosis in at least six individuals, not including those with rib lesions only that may well have been afflicted too. Given that skeletal changes only develop in 1–5% of those suffering from tuberculosis [52] this suggests a very high prevalence among the detainees.

Today, as in the past, tuberculosis disproportionately affects the poor and socially disadvantaged. On the one hand, they are more exposed to factors that reduce the resistance to infection, such as smoking, alcoholism, diabetes, silicosis, polluted air and various diseases. On the other hand, they face more risk of pathogen exposure due to crowded housing conditions, inadequate ventilation and the like [73]. Special risk groups therefore include prisoners and the homeless [96]. From about 1880 onwards tuberculosis reached epidemic proportions in Switzerland, especially among the poor [97]. While it is not possible to determine whether the illness was contracted at the institution or outside, the detainees in Cazis/Realta were undoubtedly a high-risk group for tuberculosis as they were not only poor and marginalized but also lived among many people at the institution. The high prevalence of tuberculosis and possibly other pulmonary diseases can therefore be interpreted as result of their low socioeconomic status and probably also of detention.

### Interpretation of skeletal deformities

Craniosynostosis is the premature closure of cranial sutures. If it occurs during cranial growth, the growth pattern of the skull can be altered, resulting in various deformations. Most commonly, craniosynostosis occurs as an isolated phenomenon, but it can also be associated with syndromes [52]. In two cases of craniosynostosis, there was no further skeletal evidence of a syndrome, but the mild plagiocephaly in one male may be syndromatic (g20, see below).

Both the reduced size and appearance of the skull of one male (g69, Fig 17) were characteristic for a case of microcephaly [52,73]. The possible causes include an infection of the mother during pregnancy (for example with rubella or toxoplasmosis), foetal alcohol syndrome, the interruption of blood supply to the foetal brain as well as chromosomal changes or some very rare syndromes. Microcephaly is often associated with significant cognitive disability and sometimes also with loss of hearing and vision [98], and it is a likely reason for this man being institutionalised.

Scoliosis, which was found in four individuals, occurs in association with syndromes and malformations or can be acquired, for example as a result of an infectious disease such as tuberculosis and poliomyelitis [99]. About 80–90% of all cases are idiopathic, although a hereditary background is suspected [52]. Narrowing down possible causes based on skeletal remains is difficult, but because no associated malformations indicative of syndromes or signs of infectious disease in the spines were found, they were most likely idiopathic. In more severe cases, symptoms such as back and neck pain as well as headache are expected. Pulmonary and cardiac function may be impaired as a result of the deformation and compression of the thorax [100].

Hip deformities that were found in four individuals can have different causes. Legg-Calvé-Perthes disease is a mostly unilateral aseptic necrosis of the femoral epiphysis which usually occurs between the ages of five and seven years as a result of a circulatory disorder [100]. Another possible cause is a slipped femoral capital epiphysis that mostly occurs in boys from the age of nine years. It is bilateral in about 25% of all cases and is probably linked to hormonal factors [52,100]. After healing, it is recognized by a dislocation of the centre of the head to the axis of the neck. In both Legg-Calvé-Perthes disease and slipped epiphysis, an early onset of severe degenerative changes is typical [54]. Slipped epiphysis was ruled out in all cases because the position of the femoral head was normal. One bilateral case was attributed to a syndrome (g20, see below). Severe degenerative changes in the affected joint were noted only in one young individual (g56), so that Legg-Calvé-Perthes disease was considered a possible diagnosis. In the two remaining cases, the bilateral occurrence (Fig 18) and less pronounced degenerative changes ruled out this diagnosis. These individuals also exhibited other deformities. In one (g26), the skull appeared to be extremely brachycephalic (short, broad), the humeri were squat and angled and the individual’s stature was the shortest recorded in the male sample with 151.4 cm [101]. The stature of 162.9 cm for the other individual (g88) was also below the mean of 169.1 cm for the males in the assemblage, and the lateral maxillary incisor was not erupted. Because the alpine regions of Switzerland (including Grisons) are regions of iodine deficiency and recorded a high prevalence of endemic hypothyroidism in earlier centuries, this is a possible cause [102]. The variability of skeletal manifestations of congenital hypothyroidism is considerable. Known skeletal manifestations include reduced stature, reduced diaphyseal diameter, various dysplasias (especially femoral head), delayed fusion of epi- and apophyses, brachycephalization, reduced cranial capacity, abnormal *foramen magnum*, enlarged *sella turcica*, and abnormal number and position of teeth. Flat, mushroom-shaped femoral heads with short necks are reported frequently [54,102]. Based on these criteria, endemic hypothyroidism was considered a plausible cause of the malformations in two cases (g26, g88). The expected symptoms such as mental retardation, deafness or speech disorders could be the cause of detention at the institution.

Through an elimination process a syndrome consistent with the skeletal changes in a male with a combination of several malformations (g20, Fig 19) was identified. Stickler syndrome (hereditary progressive arthro-ophthalmopathy) is an autosomal dominant disorder of connective tissues which is caused by the mutation of various collagen genes [103]. In the skull, flattened facial features due to hypoplastic facial bones, a broad and flat nose ridge as well as cleft palates are characteristic, all of which were identified in the individual from Cazis/Realta. In 94% of adults with Stickler syndrome, the vertebral endplates are altered, 80% exhibit Schmorl’s nodes and 54% develop a kyphosis of the Morbus Scheuermann type [104]. These changes were also observed in the skeleton. In joints, epiphyseal dysplasia and moderate hypermobility are characteristic. The hip joint is often affected by chondrolysis and Legg-Calvé-Perthes disease, Coxa valga, and premature degenerative changes that are believed to be the result of dysplasia, hypermobility, and articular cartilage defects [105,106]. The changes in the shoulder joints of the individual corresponded to those that have been described in cases of subluxations [107], and the disintegration and degenerative alterations of the hip joint, as well as in the hand and foot bones, are possible consequences of joint hypermobility or articular cartilage defects. A system utilising weighted criteria has been developed for the diagnosis of this syndrome [108]. Even though only the skeletal changes could be assessed, enough criteria were fulfilled to justify a diagnosis of Stickler syndrome. Progressive myopia is characteristic in this syndrome, as are cataracts, glaucoma, and retinal detachments. Therefore, the individual may have been visually impaired if not blind. The hearing is often affected as well [105]. It is likely that the syndrome was the cause of this man’s detention.

### Trauma

#### Causes of the traumata in the assemblage

The most common causes of mandibular fractures in men are assaults, especially fistfights. Other common causes include bicycle accidents and falls [109,110]. The incompletely healed mandibular fracture (g10, Fig 9) is therefore suggestive of an incident of interpersonal violence at the institution, even more so because the same individual exhibited an ulnar shaft fracture (Fig 11) and rib fractures in the same stage of healing. The healed blunt force injury in another male (g71, Fig 10) is also indicative of interpersonal violence. In penetrating brain injuries and all forms with cortical haemorrhage, there is a particularly high risk of posttraumatic epilepsy attributed to a chronic epileptogenic focus [111]. A bone fragment protruding inward is one of the most obvious triggers of post-traumatic epilepsy [112]. While the blunt force injury could have been incurred outside the institution, the possible posttraumatic epilepsy may have led to the man being institutionalized.

Clavicular fractures result from direct or indirect trauma, mostly from falls [113]. All were completely healed, except one case. Distal fractures of the radius from falling on the outstretched hand, all of which were completely healed, are the most common fracture, especially in elderly women [114]. Ulnar shaft fractures that were healed in two out of three cases, often result from falls from a height or from parrying a blow, which is why they are often considered an indicator of interpersonal violence [56]. Fractures of the olecranon can be avulsion injuries, but this case was an oblique fracture and thus more indicative of hyperextension of the elbow [113]. Both individuals with femoral neck fractures, one of which was incompletely healed (Fig 12), were elderly and were diagnosed with pathological bone loss. They therefore correspond to the typical group most at risk of this fracture type that can occur due to trivial incidents like falls after tripping [115]. Fractures of the tibia/fibula can be caused by direct or indirect forces such as blows, falls from a height or forced rotation of the foot [115]. All cases were completely healed.

Other postcranial fractures affected all bones except the sternum and the sacrum. «Clay shoveller’s fractures» that were found in two males are activity-related and mostly occur as avulsion injuries from sudden contraction of the rhomboid muscles, often in males who take up unfamiliar physical labour like shovelling, and more rarely as result of a blow [116,117]. It is possible that they resulted from forced labour. The incompletely healed compression fractures in three males may have been caused by falls from a height [117], and the remaining cases were identified as pathological fractures due to bone loss.

Rib fractures usually result from direct blunt impacts to the chest. The leading causes today are motor vehicle accidents, falls and assaults. Occasionally they also occur as pathological fractures in osteoporosis, osteomalacia and continuous coughing [117,118]. Rib fractures are discussed in more detail below.

Scapular fractures in adults are usually the result of a direct blow and are often associated with clavicular or rib fractures [113]. Nowadays, scapular fractures mostly occur in motor vehicle accidents, falls from a height and assaults [119]. Bilateral fractures of the body have also been reported in epileptic seizures and electrocution [120]. So-called pseudofractures that can progress to full fractures following minimal trauma are characteristic for osteomalacia. In the scapula, they are known to typically occur at the lateral border (Fig 14C). Further locations of such pseudofractures include the pubic rami, the medial femoral neck and medial sub-trochanteric region and the ribs [121]. The scapular fractures as well as their associated fractures in ribs, the femoral neck and the inferior pubic ramus in four individuals (g38, g48, g51, g59) are consistent with stress fractures reported in osteomalacia [121,122]. The remaining scapular fractures in males more likely represent traumatic fractures. In one male, who may have been affected by posttraumatic epilepsy (g71), seizures could be considered as a possible cause for the scapular fracture (Fig 14B). The perimortem scapular and rib fractures in a male (g3, Figs 14A and 15A) most likely occurred simultaneously in the same incident, perhaps a heavy blow to the back or a fall from a height.

The fractures of hand bones were all completely healed except a spiral fracture of a fifth metacarpal. Fractures of hand bones are mostly the result of a fall but they can also be suggestive of interpersonal violence, especially when they affect the fifth metacarpal [113].

The incompletely healed pubic fractures affected two elderly females. These fractures mostly occur from simple falls in the elderly with women being affected more frequently [117,123], or as stress fractures in osteomalacia [121] which was identified as the likely cause in one case. The acetabular and pelvic fracture in a male (g98) was completely healed but resulted in severe secondary alterations that certainly led to mobility problems which may have led to this man being institutionalized.

Patellar fractures, one of which was identified in the sample, result from falls or blows to the knee [115]. The completely healed cases of articular impression fractures of the talus and calcaneus were consistent with falls from a height [73], the incompletely healed phalangeal fracture of a small toe is a common occurrence when stumping the toe [115]. Lastly there was a case of an amputated big toe, but it was not possible to determine whether this was done intentionally or accidentally.

#### Healed trauma: Occupation and socioeconomic class

The traumata in this assemblage can shed light on incidents both outside and inside the institution. Given the long average duration of detention it seems likely that incompletely healed fractures (<6 months before death) were sustained at the institution, and completely healed fractures were more likely to have been incurred outside. This will be used as a simplifying assumption for the following considerations.

Most long bone fractures were completely healed (Fig 16) and could be attributed to accidental causes. A distinct sex-related difference was found in their patterning. The findings in Cazis/Realta were very similar to those in the lower socioeconomic class cemetery in Bern (Fig 13). The long bone fracture rates in the two assemblages from Bern that represented a higher and a lower social stratum were significantly different, but only in the male subsamples. Furthermore, fractures of the tibia and fibula were only found in the lower class male sample [58]. While the overall fracture rate in the Cazis/Realta assemblage is high for the time (Table 9), it cannot be considered unusual. Instead, it appears to reflect the predominance of males in the sample and the fact that detainees were usually from the lower social class. All in all, the long bone fractures most likely reflect the accident risks encountered in daily life by a lower socioeconomic class population in this epoch. Other completely healed postcranial fractures in the sample could also be attributed to accidental causes in most cases.

#### Trauma during detention: Underlying diseases, occupation and interpersonal violence

The prevalence, spectrum and patterning of incompletely and completely healed traumata should not differ greatly from each other and from the findings in regular cemeteries if the injuries were mainly the result of falls and occupation-related accidents. This was not the case. Few incompletely healed long bone fractures and other typically accident-related fracture types were found. However, scapular and rib fractures were found in numbers that not only by far exceeded the numbers in regular cemetery populations (Table 10), but a large proportion were also incompletely healed (Fig 16). To identify reasons as to why so many scapular and rib fractures were sustained during detention, underlying diseases, forced labour and interpersonal violence are discussed.

Though there is no objective method of diagnosing this in skeletal remains [81], the pronounced bone loss in eleven individuals was suggestive of osteoporosis or osteomalacia [121]. In primary osteoporosis, bone atrophy is seen mostly in women postmenopausally (Type I), while Type II (senile osteoporosis) is seen in both sexes and is related to increasing age [124]. In secondary osteoporosis bone loss is related to another condition such as alcoholism, hypogonadism, hormonal problems, diseases of the gastrointestinal tract or liver or calcium deficiency [125,126]. Age is the most common underlying factor in osteoporosis, but sedentary lifestyle, immobilization, smoking, genetic predisposition and various other factors also play a role [124,125]. Osteomalacia from vitamin D deficiency results in a softening and weakening of the skeleton, followed by generalised deformities as well as Looser’s zones and fractures. Causes for vitamin D deficiency are inadequate cutaneous exposure to sunlight, malnutrition and some relatively rare intestinal malabsorption syndromes [121]. Based on characteristic stress fractures, possible osteomalacia was diagnosed in three individuals for which considerable bone loss had been noted, and one in which this was not the case. The remaining individuals with bone loss that was attributed to osteoporosis comprised three females and five males. The fairly large number of osteoporotic males in this sample may be related to causes for detention, for example alcoholism that could have led to secondary osteoporosis.

Altogether, the individuals affected by bone loss accounted for all or most of the vertebral, femoral neck and pelvic as well as several scapular fractures. They raised the overall fracture rate only insignificantly, but osteomalacia clearly did contribute to the high prevalence of scapular fractures. Such fracture types were not found in any of the regular cemetery assemblages, indicating that osteomalacia was a specific problem in this group. However, these underlying diseases can still not explain the remaining scapular fractures and the very high prevalence of incompletely healed rib fractures, even more so because elderly individuals with bone loss are represented in regular cemetery samples as well.

The fracture risk of individuals with epilepsy is about twice the risk of the overall population, with the pelvis and the femur being particularly affected [127,128]. It is uncertain though whether this is the result of more frequent falls or of bone demineralisation caused by long-term use of antiepileptic medicines that are available today [127,129]. Even though epileptics were detained in Cazis/Realta, it seems unlikely that this would have greatly influenced the findings. Firstly, they were few and secondly, they were not for certain buried in this cemetery.

Forced labour, which many detainees were subjected to, could have also led to an increased fracture risk. In this case, the prevalence of accident-related fractures which are also found in regular cemeteries would be expected to be elevated, because the work at the institution probably did not differ much from physical labour in the normal population. In 1860 around 70% of the population of Grisons worked in agriculture and forestry [130]. Most of the typical accident-related fractures in the Cazis/Realta sample, however, are completely healed and less likely to have been sustained during detention.

Skeletal indicators of interpersonal violence include skull injuries, rib and scapular trauma as well as defence injuries to the forearm and hands [73]. The incompletely healed fractures of a mandible (Fig 9), a fifth metacarpal and an ulna (Fig 11) fit into this category. Multiple rib fractures in different stages of healing are generally suggestive of interpersonal violence and abuse, e.g. of children or elders [56,131,132]. In documented cases of recent human rights violations in Kosovo and Peru the most common traumata were multiple rib fractures in different stages of healing, often at the vertebral or sternal rib end. The assaults that had led to these injuries comprised punches and kicks to the thorax and stepping on the victim’s back [133].

The abundance of rib fractures in different stages of healing, often at the vertebral or sternal end in the assemblage, corresponds to this picture. Scapular fractures are often the result of assaults [73,134] and the cases that were not attributed to osteomalacia or possibly epilepsy may have been caused by blows or kicks to the back.

Blunt chest trauma is often accompanied by other injuries such as pulmonary contusion, pneumothorax and hemothorax, which can occur several days after the trauma. [133]. The mortality and morbidity of older people who suffer from a blunt chest trauma with rib fractures is twice as high as in younger people with comparable injuries. Any additional rib fracture in the elderly increases mortality by 19% and the risk of pneumonia by 27% [135]. It can be assumed that the numerous incompletely healed rib fractures were often related to the death of detainees.

The completely healed rib fractures also by far exceeded the numbers found in the reference groups. Explanations for this include a perhaps occupation-related higher risk of rib fractures in this group, but also the long average duration of detention and the fact that many detainees were admitted to the facility multiple times. It is therefore possible that some of the healed fractures were also incurred during detention.

After excluding other causes, the extremely numerous rib fractures in inmates of the psychiatric hospital in Hall were also mainly attributed to interpersonal violence [31]. Violence between inmates or between staff and inmates in psychiatric institutions, but also in other institutional contexts, is a problem across time [136,137]. Previous work by historians [4,9] has not specifically explored the topic of violence at institutions of administrative detention in the canton of Grisons, but it is known that the detainees at a workhouse in the neighbouring Canton of St. Gallen had repeatedly complained about being beaten, kicked and verbally assaulted by the wardens. Furthermore, archaic punishments such as chaining were used well into the 20^th^ century [1]. Whether violence was as widespread in other types of administrative detention facilities in Switzerland is unknown. It is possible that the combination of a correctional facility with an asylum in Cazis/Realta may have exacerbated the problem. The examination of other skeletal assemblages may shed light on this. Altogether it can be concluded from the findings that trauma contributed significantly to the mortality of the detainees.

## Conclusions

This is the first study to assess the physical causes and effects of administrative detention at a 19^th^/20^th^ century correctional facility/workhouse and asylum in Switzerland and to explore how they are interwoven. A comprehensive palaeopathological examination of 103 skeletons showed that ill health due to pre-existing conditions and the socioeconomic background contributed to the chance of being detained, and that detention led to further deterioration of health. Detainees were at higher risk of infectious disease, especially tuberculosis, to which both poverty and detention likely contributed. Scurvy and osteomalacia were unusually frequent in this group. It was not possible to determine whether these deficiency diseases occured as a result of detention or if they were linked to causes of detention such as poverty or alcoholism. However, osteomalacia clearly did contribute to increased rates of specific fractures incurred during detention. The prevalence and patterning of incompletely healed trauma that was not linked to underlying diseases was suggestive of widespread interpersonal violence at the facility.

The examination of human remains offers insights that could not be gained from written records alone and can therefore add valuable aspects to the confrontation of a system that is today seen as unethical, and the process of coming to terms with this past. While there are written records from this early phase of administrative detention in Switzerland, the persons concerned are no longer alive to bear witness to their experiences. This enhances the importance of the skeletal remains as an additional source of information.

## Supporting information

S1 Table Overview of demographic and palaeopathological findings. (PDF)

S2 Table Periosteal reactions per individual and bone. (PDF)

S3 Table Trauma per individual and bone. (PDF)

S4 Table Rib fractures per individual. (PDF)
