# Supplementary material for: Evidence of infectious disease, trauma, disability and deficiency in skeletons from the 19th/20th century correctional facility and asylum «Realta» in Cazis, Switzerland
Source: PLoS One. 2019 May 8;14(5):e0216483. doi: 10.1371/journal.pone.0216483 (PMC6505939; doi:10.1371/journal.pone.0216483)
Supplement: S1 Table — (PDF) [file pone.0216483.s001.pdf]

Table S1: Overview of demographic and palaeopathological findings.

Grave	Sex	Age	Ectocranial lesions	Endocranial lesions	Rib lesions	Vertebral lesions	Other periosteal reactions/lytic lesions	Trauma stage 1-3	Trauma stage 4	Malformations	Diagnosis
1	male	50–65		X							
2	male	45–60								X	Scoliosis
3	male	40–55						X	X	X	Craniosynostosis
4	male	55–70									
5	male	50–65							X		
6	male	40–55									
7	male	55–70					X		X		
8	male	30–45									
9	male	45–60					X				
10	male	45–60						X	X		
11	male	40–55			X		X				
12	male	40–55									
13	female	45–60									
14	female	40–55			X						
15	male	50–65							X		
16	female	40–55			X				X		
17	male	55–70									
18	male	30–45						X	X		Osteoporosis
19	male	25–40									
20	prob. male	50–65		X	X		X	X		X	Stickler Syndrome, tuberculosis
21	male	45–60							X		
22	female	45–60									
23	female	40–55					X	X			Tuberculosis?
24	male	40–55			X						
25	male	20–30			X						
26	male	40–55								X	Cretinism?
27	female	40–55									
28	male	60–75					X				
29	male	50–65						X	X		
30	female	50–65			X						
31	male	55–70									
32	female	25–40									
33	female	40–55									
34	female	50–65									
35	male	45–60					X	X	X		
36	male	40–55				X		X	X		
37	female	25–35							X		
38	female	50–65						X	X		Osteomalacia
39	male	40–55							X	X	Femoral anteversion
40	female	45–60			X		X	X	X		
41	male	40–55			X		X	X		X	Scoliosis, tuberculosis
42	female	45–60							X		
43	male	55–70						X	X		
44	female	60–75						X	X		Osteoporosis
45	female	35–50			X			X	X		
46	female	35–50			X						
47	female	40–55	X								Scurvy
48	female	55–70					X	X	X		Osteomalacia
49	female	18–21	X								
50	male	25–40				X			X		
51	female	55–70						X	X		Osteomalacia
52	female	40–55						X			
53	male	45–60							X	X	Scoliosis
54	female	25–35			X						
55	prob. male	14–17				X					Tuberculosis
56	male	20–30							X	X	Legg-Calvé-Perthes disease?
57	female	45–60						X			
58	male	65–80				X					
59	male	30–45						X			Osteomalacia
60	female	50–65									Osteoporosis

Grave	Sex	Age	Ectocranial lesions	Endocranial lesions	Rib lesions	Vertebral lesions	Other periosteal reactions/lytic lesions	Trauma stage 1-3	Trauma stage 4	Malformations	Diagnosis
61	male	20–25	X		X		X				Congenital syphilis, scurvy?
62	male	50–65							X		
63	male	45–60						X			
64	female	45–60									
65	male	50–65					X		X		
66	male	60–75							X		
67	female	35–50	X	X			X				Tuberculosis
68	male	40–55				X			X	X	Craniosynostosis, osteoporosis
69	male	40–55	X		X					X	Microcephaly
70	male	55–70							X		
71	male	50–65						X	X		Posttraumatic epilepsy?
72	female	55–70						X			
73	male	40–55							X		
74	female	30–45			X				X		
75	prob. female	30–45	X	X	X			X			Tuberculosis
76	male	40–55	X						X		Scurvy
77	female	45–60									
78	male	50–65						X	X	X	Osteoporosis, femoral anteversion
79	female	45–60									
80	male	60–75						X	X	X	Craniosynostosis, osteoporosis
81	male	55–70						X	X		
82	male	50–65						X			
83	male	55–70					X		X		
84	male	55–70		X			X	X	X		
85	female	45–60			X			X	X		
86	male	50–65									
87	male	55–70						X	X		
88	male	50–65								X	Cretinism?
89	male	60–75							X		
90	prob. male	25–40	X				X			X	Scoliosis, scurvy
91	male	50–65		X			X	X	X		
92	female	40–55					X		X		
93	male	25–40									
94	male	35–50									
95	male	40–55						X			
96	male	40–55				X					Tuberculosis
97	male	35–50									
98	male	60–75							X		Protrusio acetabuli (posttraumatic)
99	male	50–65				X			X		
100	male	45–60			X						Osteoporosis
101	male	45–60							X		
102	female	20–30	X								Scurvy
103	male	50–65							X		
