# Supplementary material for: Evidence of infectious disease, trauma, disability and deficiency in skeletons from the 19th/20th century correctional facility and asylum «Realta» in Cazis, Switzerland
Source: PLoS One. 2019 May 8;14(5):e0216483. doi: 10.1371/journal.pone.0216483 (PMC6505939; doi:10.1371/journal.pone.0216483)
Supplement: S3 Table — (PDF) [file pone.0216483.s003.pdf]

Table S3: Trauma per individual and bone.

Grave	Sex	Age min.	Age max.	Frontal	Parietal left	Parietal right	Occipital	Temporal left	Temporal right	Sphenoid left	Sphenoid right	Facial bones left	Facial bones right	Mandible	Clavicula left	Clavicula right	Humerus left	Humerus right	Radius left	Radius right	Ulna left	Ulna right	Femur left	Femur right	Tibia left	Tibia right	Fibula left	Fibula right	Cervical vertebrae	Thoracic vertebrae	Lumbar vertebrae	Scapula left	Scapula right	Sternum	Ribs left	Ribs right	Hand left	Hand right	Pelvis left	Pelvis right	Sacrum	Patella left	Patella right	Foot left	Foot right
1	male	50	65	0	0	0	1	0	0.5	0	0	0	0	1	1	1	1	1	1	1	1	1	1	1	1	1	1	1	1	1	1	1	1	0	1	1	1	1	1	1	1	1	1	1	1
2	male	45	60	0	0	0	0	0	0	0	0	0	0	0	0	0	0.5	0	1	1	1	1	1	1	1	1	1	1	0	1	1	1	1	0.5	1	1	1	1	1	1	1	1	1	1	1
3	male	40	55	1	1	1	1	1	1	1	1	1	1	1	1	1	1	1	1	1	1	1	1	1	1	1	1	1	1	1	1	1	1	1	1	1	1	1	1	1	1	1	1	1	1
4	male	55	70	1	1	1	1	1	1	1	0	0	0	1	0.5	0.5	1	1	1	1	1	1	1	1	1	1	1	1	0	0.5	1	0	0	1	0	0	0.5	1	1	1	1	1	1	1	1
5	male	50	65	1	1	1	1	1	1	1	1	1	1	1	1	1	1	1	1	1	1	1	1	1	1	1	1	1	1	1	1	1	1	1	1	1	1	1	1	1	1	1	1	1	1
6	male	40	55	1	1	1	1	1	1	1	1	1	1	1	1	1	1	1	1	1	1	1	1	1	1	1	1	1	1	1	1	1	1	1	1	1	1	1	1	1	1	1	1	1	1
7	male	55	70	1	1	1	1	1	1	1	1	1	1	1	1	1	1	1	1	1	1	1	1	1	1	1	1	1	1	1	1	1	1	1	1	1	1	1	1	1	1	1	1	1	1
8	male	30	45	1	1	1	1	1	1	1	1	1	1	1	1	1	1	1	1	1	1	1	1	1	1	1	1	1	0.5	0.5	0	0	0	0	0	0	0.5	1	0.5	0.5	0.5	1	0	0	0
9	male	45	60	1	1	1	1	1	1	1	1	1	1	1	1	1	1	1	1	1	1	1	1	1	1	1	1	1	1	1	1	1	1	1	1	1	1	1	1	1	1	1	1	1	1
10	male	45	60	1	1	1	1	1	1	1	1	1	1	1	1	1	1	1	1	1	1	1	1	1	1	1	1	1	1	1	1	1	1	1	1	1	1	1	1	1	1	1	1	1	1
11	male	40	55	1	1	1	1	1	1	1	1	1	1	1	1	1	1	1	1	1	1	1	1	1	1	1	1	1	1	1	1	1	1	1	1	1	1	1	1	1	1	1	1	1	1
12	male	40	55	1	1	1	1	1	1	1	1	1	1	1	1	1	1	1	1	1	1	1	1	1	1	1	1	1	1	1	1	1	1	1	1	1	1	1	1	1	1	1	1	1	1
13	female	45	60	1	1	1	1	1	1	1	1	1	1	1	1	1	1	1	1	1	1	1	1	1	1	1	1	1	0	0.5	0.5	0.5	0.5	0.5	0.5	0.5	1	1	0.5	0.5	0	1	1	0.5	0.5
14	female	40	55	1	1	1	1	1	1	1	1	0	0	1	1	1	1	1	1	1	1	1	1	1	1	1	1	1	0.5	1	1	0.5	0.5	0.5	0.5	0.5	0	0	1	1	1	1	1	0.5	1
15	male	50	65	1	1	1	1	1	1	1	1	1	1	1	1	1	1	1	1	1	1	1	1	1	1	1	1	1	1	0	0	0	0	0.5	0	0	1	1	0	0	0	1	1	1	1
16	female	40	55	1	1	1	1	1	1	1	1	1	1	1	1	1	1	1	1	1	1	1	1	1	1	1	1	1	1	1	1	1	1	1	1	1	1	1	1	1	1	1	1	1	1
17	male	55	70	1	1	1	1	1	1	1	1	1	1	1	1	1	1	1	1	1	1	1	1	1	1	1	1	1	1	1	1	1	1	0.5	1	1	1	1	1	1	1	1	1	1	1
18	male	30	45	1	1	1	1	1	1	1	1	1	1	1	1	1	1	1	1	1	1	1	1	1	1	1	1	1	1	1	1	1	1	1	1	1	1	1	1	1	1	1	1	1	1
19	male	25	40	1	1	1	1	1	1	1	1	1	1	1	1	1	1	1	1	1	1	1	1	1	1	1	1	1	1	1	1	1	0.5	1	1	1	1	1	1	1	1	1	1	1	1
20	prob. male	50	65	1	1	1	1	1	1	1	1	1	1	1	1	1	1	1	1	1	1	1	1	1	1	1	1	1	1	1	1	1	1	1	1	1	1	1	1	1	1	1	1	1	1
21	male	45	60	0.5	1	1	1	1	0.5	0	0	0	0	1	1	1	1	1	1	1	1	1	1	1	1	1	1	1	0.5	1	1	1	1	1	1	1	1	1	0.5	0.5	1	1	1	1	1
22	female	45	60	1	1	1	1	1	1	1	1	1	1	1	1	1	1	1	1	1	1	1	1	1	1	1	1	1	1	1	1	1	1	1	1	1	1	1	1	1	1	1	1	0	0
23	female	40	55	1	1	1	1	1	1	1	1	1	1	1	1	1	1	0.5	0.5	0	1	0.5	0	1	0	0	1	1	1	0	0	0	0	0	0	0.5	1	1	0	0	0	1	0.5	0	0.5
24	male	40	55	1	1	1	1	1	1	1	1	1	1	1	1	1	1	1	1	1	1	1	1	1	1	1	1	1	1	1	1	1	1	1	1	1	1	1	1	1	1	1	1	1	1
25	male	20	30	0	0	0	0	0	0	0	0	0	0	1	1	1	1	0	1	1	1	1	1	1	1	1	1	1	1	1	1	0	1	1	1	1	1	1	1	1	1	1	1	1	1
26	male	40	55	1	1	1	1	1	1	1	1	1	1	1	1	1	1	1	1	1	1	1	1	1	1	1	1	1	0	0	0.5	0.5	1	0	0.5	0.5	1	1	1	1	1	1	1	1	1
27	female	40	55	1	1	1	1	1	1	1	1	1	1	1	1	1	1	1	1	1	1	1	1	1	1	1	1	1	1	1	1	1	1	1	1	1	1	1	1	1	1	1	1	1	1
28	male	60	75	1	1	1	1	1	1	1	1	1	1	1	1	1	1	1	1	1	1	1	1	1	1	1	1	1	1	1	1	1	1	0	1	1	1	1	1	1	1	1	1	0.5	0.5
29	male	50	65	1	1	1	1	1	1	1	1	1	1	1	1	1	1	1	1	1	1	1	1	1	1	1	1	1	1	1	1	1	1	1	1	1	1	1	1	1	1	1	1	1	1
30	female	50	65	1	1	1	1	1	1	1	1	1	1	1	1	1	1	1	1	1	1	1	1	1	1	1	1	1	1	1	1	1	1	1	1	1	1	1	1	1	1	1	1	1	1
31	male	55	70	1	1	1	1	1	1	1	1	1	1	1	1	1	1	1	1	1	1	1	1	1	1	1	1	1	1	1	1	1	1	0	1	1	1	1	1	1	1	1	1	1	1
32	female	25	40	1	1	1	1	1	1	1	1	1	1	1	1	1	1	1	1	1	1	1	1	1	1	1	1	1	1	1	1	1	1	1	1	1	1	1	1	1	1	1	1	1	1
33	female	40	55	1	1	1	1	1	1	0	0	0	0	0	0	0	1	0.5	0.5	0	0.5	0	1	1	1	1	0.5	0.5	0	0	0	0	0	0	0	0	0	0	0	0	0	0	0	0	0
34	female	50	65	1	1	1	1	1	1	1	1	1	1	1	1	1	1	1	1	1	1	1	1	1	1	1	1	1	1	1	1	1	1	1	1	1	1	1	1	1	0.5	1	1	1	1
35	male	45	60	1	1	1	1	1	1	1	1	0.5	0.5	1	1	1	1	1	1	1	1	1	1	1	1	1	1	1	1	1	1	1	1	1	1	1	1	1	1	1	1	1	1	1	1
36	male	40	55	1	1	1	1	1	1	1	1	1	1	1	1	1	1	1	1	1	1	1	1	1	1	1	1	1	1	1	1	0	0.5	0	0.5	0.5	1	1	0.5	0.5	0.5	1	1	1	1
37	female	25	35	1	1	1	1	1	0.5	0.5	0	0	0	0.5	1	1	1	1	1	1	1	1	1	1	1	1	1	1	0	0	0.5	0	0	0	0	0	0.5	0	0.5	0.5	1	1	1	1	1
38	female	50	65	1	1	1	1	1	1	1	1	1	1	1	1	1	1	1	1	1	1	1	1	1	1	1	1	1	1	0	0	1	1	0	0.5	0.5	1	1	0.5	0	0	1	1	1	1
39	male	40	55	1	0.5	0	1	1	1	1	1	1	1	1	1	1	1	1	1	1	1	1	1	1	1	1	1	1	1	1	1	1	1	1	1	1	1	1	1	1	1	1	1	1	1
40	female	45	60	1	1	1	1	1	1	1	1	1	1	1	1	1	1	1	1	1	1	1	1	1	1	1	1	1	1	1	1	1	1	1	1	1	1	1	1	1	1	1	1	1	1
41	male	40	55	1	1	1	1	1	1	1	1	1	1	1	1	1	1	1	1	1	1	1	1	1	1	1	1	1	1	1	1	1	1	1	1	1	1	1	1	1	1	1	1	1	1
42	female	45	60	1	1	1	1	1	1	1	1	1	1	1	1	1	1	1	1	1	1	1	1	1	1	1	1	1	1	1	1	1	1	1	1	1	1	1	1	1	1	1	1	1	1
43	male	55	70	1	1	1	1	1	1	1	1	1	1	1	1	1	1	1	1	1	1	1	1	1	1	1	1	1	1	1	1	1	1	1	1	1	1	1	1	1	1	1	1	1	1
44	female	60	75	1	1	1	1	1	1	1	1	1	1	1	1	1	1	1	1	1	1	1	1	1	1	0.5	1	0.5	1	1	1	1	1	0	1	1	1	1	1	1	1	1	1	1	1
45	female	35	50	1	1	1	1	1	1	1	1	1	1	1	1	1	1	1	1	1	1	1	1	1	1	1	1	1	1	1	1	1	1	1	1	1	1	1	1	1	1	1	1	1	1
46	female	35	50	1	1	1	1	1	1	1	1	1	1	1	1	1	1	1	1	1	1	1	1	1	1	1	1	1	1	1	1	1	0.5	1	1	1	1	1	1	1	1	1	1	1	1
47	female	40	55	1	1	1	1	1	1	1	1	1	1	1	1	1	1	1	1	1	1	1	1	1	1	1	1	1	1	1	1	1	0.5	0.5	1	1	1	1	1	1	1	1	1	1	1
48	female	55	70	1	0.5	0	0	1	0	1	1	1	1	1	0.5	0.5	1	1	1	1	1	1	1	1	1	1	1	1	0.5	1	1	1	1	1	1	1	1	1	1	1	1	1	1	1	1
49	female	18	21	1	1	1	1	1	1	1	1	1	1	1	1	1	1	1	1	1	1	1	1	1	1	1	1	1	0	0	1	0.5	0.5	0	0.5	0.5	1	1	1	1	1	1	1	1	1
50	male	25	40	1	1	1	1	1	1	1	1	1	1	1	1	0.5	1	1	1	1	1	1	1	1	1	1	1	1	0.5	1	1	1	1	1	1	0.5	1	1	1	1	1	1	1	1	1
51	female	55	70	1	1	1	1	1	1	1	1	1	1	1	1	1	1	1	1	1	1	1	1	1	1	1	1	1	1	1	1	1	1	1	1	1	1	1	1	1	1	1	1	1	1
52	female	40	55	1	1	1	1	1	1	1	1	1	1	1	1	1	1	1	1	1	1	1	1	1	1	1	1	1	1	1	1	1	1	1	1	1	1	1	1	1	1	1	1	1	1
53	male	45	60	1	1	1	1	1	1	1	1	1	1	1	1	1	1	1	1	1	1	1	1	1	1	1	1	1	1	1	1	1	1	1	1	1	1	1	1	1	1	1	1	1	1
54	female	25	35	1	1	1	1	1	1	1	1	1	1	1	1	1	1	1	1	1	1	1	1	1	1	1	1	1	1	1	1	1	1	1	1	1	1	1	1	1	1	1	1	1	1

Grave	Sex	Age min.	Age max.	Frontal	Parietal left	Parietal right	Occipital	Temporal left	Temporal right	Sphenoid left	Sphenoid right	Facial bones left	Facial bones right	Mandible	Clavicula left	Clavicula right	Humerus left	Humerus right	Radius left	Radius right	Ulna left	Ulna right	Femur left	Femur right	Tibia left	Tibia right	Fibula left	Fibula right	Cervical vertebrae	Thoracic vertebrae	Lumbar vertebrae	Scapula left	Scapula right	Sternum	Ribs left	Ribs right	Hand left	Hand right	Pelvis left	Pelvis right	Sacrum	Patella left	Patella right	Foot left	Foot right
55	prob. male	14	17	1	1	1	1	1	1	1	1	1	1	1	1	1	1	1	1	1	1	1	1	1	1	1	1	1	1	1	1	1	1	1	1	1	1	1	1	1	1	1	1	1	1
56	male	20	30	1	1	1	1	1	1	1	1	1	1	1	1	1	1	1	1	1	1	1	1	1	1	1	1	1	0.5	1	0.5	1	1	1	1	1	1	1	1	1	1	1	1	1	1
57	female	45	60	1	1	1	1	1	1	1	1	1	1	1	1	1	1	1	1	1	1	1	1	1	1	1	1	1	1	1	1	1	1	1	1	1	1	1	1	1	1	1	1	1	1
58	male	65	80	1	1	1	1	1	1	1	1	1	1	1	1	1	1	1	1	1	1	1	1	1	1	1	1	1	1	1	1	1	1	1	1	1	1	1	1	1	1	1	0.5	1	1
59	male	30	45	1	1	1	1	1	1	1	1	1	1	1	1	1	1	1	1	1	1	1	1	1	1	1	1	1	1	1	1	1	1	1	1	1	1	1	1	1	1	1	1	1	1
60	female	50	65	1	1	1	1	1	1	1	1	1	1	1	1	1	1	1	1	1	1	1	1	1	1	1	1	1	1	1	1	1	1	1	1	1	1	1	1	1	1	1	1	1	1
61	male	20	25	1	1	1	1	1	1	1	1	1	1	1	1	1	1	1	1	1	1	1	1	1	1	1	1	1	1	1	1	1	1	1	1	1	1	1	1	1	1	1	1	1	1
62	male	50	65	1	1	1	1	1	1	1	1	1	1	1	1	1	1	1	1	1	1	1	1	1	1	1	1	1	1	1	1	1	1	1	1	1	1	1	1	1	1	1	1	1	1
63	male	45	60	1	1	1	1	1	1	1	1	1	1	1	1	1	1	1	1	1	1	1	1	1	1	1	1	1	1	1	1	1	1	1	1	1	1	1	1	1	1	1	1	1	1
64	female	45	60	1	1	1	1	1	1	1	1	1	1	1	1	1	1	1	1	1	1	1	1	1	1	1	1	1	1	1	1	0.5	0.5	1	1	1	1	1	1	1	1	1	1	1	1
65	male	50	65	1	1	1	1	1	1	1	1	1	1	1	1	1	1	1	1	1	1	1	1	1	1	1	1	1	1	1	1	1	1	1	1	1	1	1	1	1	1	1	1	1	1
66	male	60	75	1	1	1	1	1	1	1	1	1	1	1	1	1	1	1	1	1	1	1	1	1	1	1	1	1	1	1	1	1	1	1	1	1	1	1	1	1	1	1	1	1	1
67	female	35	50	1	1	1	1	1	1	1	1	1	1	1	1	1	1	1	1	1	1	1	1	1	1	1	1	1	1	1	1	1	1	1	1	1	1	1	1	1	1	1	1	1	1
68	male	40	55	1	1	1	1	1	1	1	1	1	1	1	1	1	1	1	1	1	1	1	1	1	1	1	1	1	1	1	1	1	1	1	1	1	1	1	1	1	1	1	1	1	1
69	male	40	55	1	1	1	1	1	1	1	1	1	1	1	1	1	1	1	1	1	1	1	1	1	1	1	1	1	1	1	1	1	1	0.5	0.5	0.5	1	1	1	1	1	1	1	1	1
70	male	55	70	1	1	1	1	1	1	1	1	0.5	1	1	1	1	1	1	1	1	1	1	1	0	1	1	1	1	1	1	1	1	1	1	1	1	1	1	1	1	1	1	0	1	1
71	male	50	65	1	1	1	1	1	1	1	1	1	1	1	1	1	1	1	1	1	1	1	1	1	1	1	1	1	1	1	1	1	1	1	1	1	1	1	1	1	1	1	1	1	1
72	female	55	70	1	1	1	1	1	1	1	1	1	1	1	1	1	1	1	1	1	1	1	1	1	1	1	1	1	1	1	1	1	1	0.5	1	1	1	1	1	1	1	0	0	1	1
73	male	40	55	1	1	1	1	1	1	1	1	1	1	1	1	1	1	1	1	1	1	1	1	1	1	1	1	1	1	1	1	1	1	1	1	1	1	1	1	1	1	1	1	1	1
74	female	30	45	1	1	1	1	1	1	1	1	1	1	1	1	1	1	1	1	1	1	1	1	1	1	1	1	1	1	1	1	1	1	1	1	1	1	1	1	1	1	0	1	1	1
75	prob. female	30	45	1	1	1	1	1	1	1	1	1	1	1	1	1	1	1	1	1	1	1	1	1	1	1	1	1	1	1	1	1	1	1	1	1	1	1	1	1	1	1	1	1	1
76	male	40	55	1	1	1	1	1	1	1	1	1	1	1	1	1	1	1	1	1	1	1	1	1	1	1	1	1	1	1	1	1	1	1	1	1	1	1	1	1	1	1	1	1	1
77	female	45	60	1	1	1	1	1	1	1	1	1	1	1	1	1	1	1	1	1	1	1	1	1	1	1	1	1	1	1	1	1	1	1	1	1	1	1	1	1	1	1	1	1	1
78	male	50	65	1	1	1	1	1	1	1	1	1	1	1	1	1	1	1	1	1	1	1	1	1	1	1	1	1	1	1	1	1	1	1	1	1	1	1	1	1	1	1	1	1	1
79	female	45	60	1	1	1	1	1	1	1	1	1	1	1	1	1	1	1	1	1	1	1	1	1	1	1	1	1	1	1	1	1	1	1	1	1	1	1	1	1	1	1	1	1	1
80	male	60	75	1	1	1	1	1	1	1	1	1	1	1	1	1	1	1	1	1	1	1	1	1	1	1	1	1	1	1	1	1	1	1	1	1	1	1	1	1	1	1	1	1	1
81	male	55	70	1	1	1	1	1	1	1	1	1	1	1	1	1	1	1	1	1	1	1	1	1	1	1	1	1	1	1	1	1	1	0	1	1	1	1	1	1	1	0.5	1	1	1
82	male	50	65	1	1	1	1	1	1	1	1	1	1	1	1	1	1	1	1	1	1	1	1	1	1	1	1	1	1	1	1	1	1	1	1	1	1	1	1	1	1	1	1	1	1
83	male	55	70	1	1	1	1	1	1	1	1	1	1	1	1	1	1	1	1	1	1	1	1	1	1	1	1	1	1	1	1	1	1	1	1	1	1	1	1	1	1	1	1	1	1
84	male	55	70	1	1	1	1	1	1	1	1	1	1	1	1	1	1	1	1	1	1	1	1	1	1	1	1	1	1	1	1	1	1	1	1	1	1	1	1	1	1	1	1	1	1
85	female	45	60	1	1	1	1	1	1	1	1	1	1	1	1	1	1	1	1	1	1	1	1	1	1	1	1	1	1	1	1	1	1	1	1	1	1	1	1	1	1	1	1	1	1
86	male	50	65	1	1	1	1	1	1	1	1	1	1	1	1	1	1	1	1	1	1	1	1	1	1	1	1	1	0	0.5	0.5	1	1	0	0.5	0.5	1	1	1	1	0.5	1	1	1	1
87	male	55	70	1	1	1	1	1	1	1	1	1	1	1	1	1	1	1	1	1	1	1	1	1	1	1	1	1	1	1	1	1	1	1	1	1	1	1	1	1	1	1	1	1	1
88	male	50	65	1	1	1	1	1	1	1	1	1	1	1	1	1	1	1	1	1	1	1	1	1	1	1	1	1	1	1	1	1	1	1	1	1	1	1	1	1	1	1	1	1	1
89	male	60	75	1	1	1	1	1	1	1	1	1	1	1	1	1	1	1	1	1	1	1	1	1	1	1	1	1	1	1	1	1	1	1	1	1	1	1	1	1	1	1	1	1	1
90	prob. male	25	40	1	1	1	1	1	1	1	1	0	0.5	0.5	0	0	1	1	1	1	1	1	1	1	1	1	1	1	0	0.5	1	0.5	1	0	0	0.5	1	1	0.5	1	1	1	1	1	1
91	male	50	65	1	1	1	1	1	1	1	1	1	1	1	1	1	1	1	1	1	1	1	1	1	1	1	1	1	1	1	1	1	1	1	1	1	1	1	1	1	1	1	1	1	1
92	female	40	55	1	1	1	1	1	1	1	1	1	1	1	1	1	1	1	1	1	1	1	1	1	1	1	1	1	1	1	1	1	1	1	1	1	1	1	1	1	1	1	1	1	1
93	male	25	40	1	1	1	1	1	1	1	1	1	1	1	1	1	1	1	1	1	1	1	1	1	1	1	1	1	0	0.5	1	0	0	0	0.5	0	0	0	1	1	1	1	1	0	0.5
94	male	35	50	1	1	1	1	1	1	1	1	1	1	1	1	1	1	1	1	1	1	1	1	1	1	1	1	1	1	1	1	1	1	1	1	1	1	1	1	1	1	1	1	1	1
95	male	40	55	1	1	1	1	1	1	1	1	1	1	1	1	1	1	1	1	1	1	1	1	1	1	1	1	1	1	1	1	1	1	0.5	1	1	1	1	1	1	0.5	1	1	1	1
96	male	40	55	1	1	1	1	1	1	1	1	1	1	1	1	1	1	1	1	1	1	1	1	1	1	1	1	1	1	1	1	1	1	0	1	1	1	1	1	1	0.5	1	1	1	1
97	male	35	50	1	0	1	1	0	1	1	1	1	1	1	1	1	1	1	1	1	1	1	1	1	1	1	1	1	0.5	1	1	1	1	1	0.5	0.5	0.5	0.5	1	1	1	1	1	1	1
98	male	60	75	1	1	1	1	1	1	1	1	1	1	1	1	1	1	1	1	1	1	1	1	1	1	1	1	1	1	1	1	1	1	0	1	1	1	1	1	1	1	1	1	1	1
99	male	50	65	1	1	1	1	1	1	1	1	1	1	1	1	1	1	1	1	1	1	1	1	1	1	1	1	1	1	0	1	0.5	0.5	0	0	0	1	1	0.5	0.5	0.5	1	1	1	1
100	male	45	60	1	1	1	1	1	1	1	1	1	1	1	1	1	1	1	1	1	1	1	1	1	1	1	1	1	1	1	1	1	1	1	1	1	1	1	1	1	1	1	1	1	1
101	male	45	60	1	1	1	1	1	1	1	1	1	1	1	1	1	1	1	1	1	1	1	1	1	1	1	1	1	0	0.5	1	0	0	0	0	0	1	1	1	1	1	1	1	0.5	0.5
102	female	20	30	1	1	1	1	1	1	1	1	1	1	1	1	1	1	1	1	1	1	1	1	1	1	1	1	1	1	1	1	1	1	1	1	1	1	1	1	1	1	1	1	1	1
103	male	50	65	1	1	1	1	1	1	1	1	1	1	1	1	1	1	1	1	1	1	1	1	1	1	1	1	1	1	1	1	1	1	0.5	1	1	1	1	1	1	1	1	1	1	1

0/0.5/1=bone count. Bones with trauma are highlighted in colour: red=healing stage 1, orange=healing stage 2/3, green=healing stage 4. When rib fractures in different stages of healing are present, only the lowest stage is depicted. Further details on rib fractures can be found in the S4Table.
