# Supplementary material for: Evidence of infectious disease, trauma, disability and deficiency in skeletons from the 19th/20th century correctional facility and asylum «Realta» in Cazis, Switzerland
Source: PLoS One. 2019 May 8;14(5):e0216483. doi: 10.1371/journal.pone.0216483 (PMC6505939; doi:10.1371/journal.pone.0216483)
Supplement: S4 Table — (PDF) [file pone.0216483.s004.pdf]

Table S4: Rib fractures per individual.

Grave	Sex	Age min.	Age max.	N ribs preserved	N ribs with fracture(s)	N fractures total	N fractures stage 1	N fractures stage 2	N fractures stage 3	N fractures stage 4
1	male	50	65	20	0	0	0	0	0	0
2	male	45	60	20	0	0	0	0	0	0
3	male	40	55	23	9	11	11	0	0	0
4	male	55	70	5	0	0	0	0	0	0
5	male	50	65	24	3	3	0	0	0	3
6	male	40	55	21	0	0	0	0	0	0
7	male	55	70	24	8	10	0	0	0	10
8	male	30	45	15	0	0	0	0	0	0
9	male	45	60	21	0	0	0	0	0	0
10	male	45	60	23	18	24	0	16	2	6
11	male	40	55	24	0	0	0	0	0	0
12	male	40	55	24	0	0	0	0	0	0
13	female	45	60	19	0	0	0	0	0	0
14	female	40	55	24	0	0	0	0	0	0
15	male	50	65	2	3	3	0	0	0	3
16	female	40	55	24	0	0	0	0	0	0
17	male	55	70	24	0	0	0	0	0	0
18	male	30	45	22	10	12	0	3	3	6
19	male	25	40	22	0	0	0	0	0	0
20	prob. male	50	65	23	1	1	0	1	0	0
21	male	45	60	23	0	0	0	0	0	0
22	female	45	60	18	0	0	0	0	0	0
23	female	40	55	14	1	1	0	1	0	0
24	male	40	55	24	0	0	0	0	0	0
25	male	20	30	24	0	0	0	0	0	0
26	male	40	55	12	0	0	0	0	0	0
27	female	40	55	24	0	0	0	0	0	0
28	male	60	75	24	0	0	0	0	0	0
29	male	50	65	24	8	9	0	2	6	1
30	female	50	65	24	0	0	0	0	0	0
31	male	55	70	24	0	0	0	0	0	0
32	female	25	40	22	0	0	0	0	0	0
33	female	40	55	0	0	0	0	0	0	0
34	female	50	65	23	0	0	0	0	0	0
35	male	45	60	24	4	5	0	2	2	1
36	male	40	55	24	16	16	0	6	6	4
37	female	25	35	2	0	0	0	0	0	0
38	female	50	65	10	20	41	0	28	0	13
39	male	40	55	24	0	0	0	0	0	0
40	female	45	60	24	6	6	0	3	1	2
41	male	40	55	24	2	2	0	2	0	0
42	female	45	60	24	1	2	0	1	0	1
43	male	55	70	24	5	6	0	3	2	1
44	female	60	75	24	2	2	0	2	0	0
45	female	35	50	22	4	5	0	1	0	4
46	female	35	50	21	0	0	0	0	0	0
47	female	40	55	22	0	0	0	0	0	0
48	female	55	70	20	17	27	0	18	0	9
49	female	18	21	14	0	0	0	0	0	0
50	male	25	40	15	0	0	0	0	0	0
51	female	55	70	24	14	18	0	15	0	3
52	female	40	55	24	12	13	0	12	1	0
53	male	45	60	24	3	3	0	0	0	3
54	female	25	35	24	0	0	0	0	0	0
55	prob. male	14	17	22	0	0	0	0	0	0
56	male	20	30	24	2	2	0	0	0	2
57	female	45	60	22	0	0	0	0	0	0
58	male	65	80	24	0	0	0	0	0	0
59	male	30	45	24	0	0	0	0	0	0
60	female	50	65	23	0	0	0	0	0	0
61	male	20	25	24	0	0	0	0	0	0
62	male	50	65	24	0	0	0	0	0	0
63	male	45	60	23	5	6	0	6	0	0
64	female	45	60	24	0	0	0	0	0	0
65	male	50	65	24	1	1	0	0	0	1
66	male	60	75	23	4	4	0	0	0	4
67	female	35	50	24	0	0	0	0	0	0
68	male	40	55	22	0	0	0	0	0	0
69	male	40	55	16	1	1	0	0	0	1
70	male	55	70	22	2	2	0	0	0	2
71	male	50	65	23	10	10	0	6	0	4
72	female	55	70	22	2	2	0	2	0	0
73	male	40	55	24	0	0	0	0	0	0
74	female	30	45	24	1	1	0	0	0	1
75	prob. female	30	45	22	1	1	0	0	1	0
76	male	40	55	24	0	0	0	0	0	0
77	female	45	60	24	0	0	0	0	0	0
78	male	50	65	24	5	8	0	0	2	6
79	female	45	60	23	0	0	0	0	0	0
80	male	60	75	24	16	28	0	0	1	27

Grave	Sex	Age min.	Age max.	N ribs preserved	N ribs with fracture(s)	N fractures total	N fractures stage 1	N fractures stage 2	N fractures stage 3	N fractures stage 4
81	male	55	70	24	0	0	0	0	0	0
82	male	50	65	24	1	1	0	0	1	0
83	male	55	70	24	20	49	0	0	0	49
84	male	55	70	24	5	5	0	0	3	2
85	female	45	60	24	5	6	0	1	3	2
86	male	50	65	13	0	0	0	0	0	0
87	male	55	70	24	12	13	0	0	5	8
88	male	50	65	24	0	0	0	0	0	0
89	male	60	75	24	5	6	0	0	0	6
90	prob. male	25	40	6	0	0	0	0	0	0
91	male	50	65	23	5	6	0	4	1	1
92	female	40	55	24	1	1	0	0	0	1
93	male	25	40	7	0	0	0	0	0	0
94	male	35	50	24	0	0	0	0	0	0
95	male	40	55	22	3	3	0	3	0	0
96	male	40	55	20	0	0	0	0	0	0
97	male	35	50	22	0	0	0	0	0	0
98	male	60	75	18	2	2	0	0	0	2
99	male	50	65	4	0	0	0	0	0	0
100	male	45	60	23	0	0	0	0	0	0
101	male	45	60	0	0	0	0	0	0	0
102	female	20	30	24	0	0	0	0	0	0
103	male	50	65	22	2	2	0	0	0	2
